# Factors Affecting Cypriot Nurses’ Roles in the Care and Education of Patients with CKD: An Interpretive Phenomenological Study

**DOI:** 10.3390/healthcare13131601

**Published:** 2025-07-03

**Authors:** Evangelos Latzourakis, Panayiotis Angelides, Marianna Diomidous, Monica Nikitara, Costas S. Constantinou

**Affiliations:** 1 Department of Health Sciences, University of Nicosia, 2417 Nicosia, Cyprusnikitara.m@unic.ac.cy (M.N.); 2 Department of Public Health, National and Kapodistrian University of Athens, 15772 Athens, Greece; 3 Department of Basic and Clinical Sciences, Medical School, University of Nicosia, 1700 Nicosia, Cyprus

**Keywords:** chronic kidney disease, nursing roles, patient education, facilitators, barriers

## Abstract

**Background**: Chronic kidney disease (CKD) affects over 10% of the global population and imposes a growing burden on healthcare systems. **Aim**: To explore nurses’ perceptions of their roles in CKD care and identify factors influencing role implementation. **Methods**: An Interpretative Phenomenological Approach (IPA) was employed, involving semi-structured interviews with 16 purposively selected nurses from all district hospitals in the Republic of Cyprus. Thematic analysis was conducted on the transcribed data. **Findings**: Nurses identified five core roles in CKD care: machine operator, holistic caregiver, bureaucratic coordinator, patient educator, and emotional supporter. These roles varied by setting. Key influencing factors included nurse training, organizational challenges, barriers to patient education, patient behavior, and nurses’ coping strategies. **Conclusions:** Nurses are essential to quality CKD care, particularly in patient education. A framework was developed to address barriers and support nurses, healthcare organizations, and patients in improving care delivery.

## 1. Introduction

Chronic kidney disease (CKD) is becoming increasingly prevalent as an emergent global public health issue that affects more than 10% of the global population, or more than 840 million people [1]. Its impact is not only clinical but also economic, contributing to high morbidity, mortality, and catastrophic health expenditures, particularly in low- and middle-income countries [2]. Globally, approximately 2 million people currently receive dialysis, with about 69–89% of them undergoing hemodialysis [3], whereas the annual global incidence is roughly a quarter million new dialysis patients each year [4].

Cyprus reports one of the highest rates of kidney failure in Europe, with an incidence of 204 new cases per million population [5], significantly exceeding figures from countries such as Austria (122 pmp) and the United Kingdom (116 pmp). Diabetes mellitus is the leading cause of CKD in the Cypriot population [6], and Cyprus has one of the highest incidences of renal replacement therapy (RRT) across 37 European countries. Diabetes mellitus is considered the primary cause of renal disease in Cyprus [6]. These indicators underscore the urgency of strengthening CKD care in Cyprus.

Nurses play a central role in delivering quality CKD care. Their responsibilities include monitoring treatment, managing complications, supporting patient adaptation, and delivering education [7]. In many countries, specialized nursing roles in nephrology have emerged, focusing on areas such as anemia management, vascular access, and pre-dialysis counseling [8,9,10,11,12]. However, despite these advancements, CKD still receives less attention than other chronic conditions like heart failure, even though its prevalence and associated costs are similarly high [13].

A critical gap in the literature exists regarding how registered nurses experience their roles in CKD care, particularly in contexts like Cyprus, where the disease burden is high but limitations persist. Understanding how nurses perceive and perform their roles and identifying the factors that facilitate or hinder effective care delivery are essential for informing targeted improvements in CKD management.

## 2. Research Questions

As a result of the literature review, it appears that there is a growing need to understand how nurses who provide care to people with CKD perceive and experience their roles. Additionally, there is a need to identify and study different factors that positively or negatively affect the implementation of nurses’ roles and determine the standards of nursing care provided in CKD care fields. Thus, the following research questions were developed:

1. **How do Cypriot nurses perceive and experience their role in caring for patients with chronic kidney disease (CKD) overall, including patient education for self-care management?**

*Exploring nurses’ perceptions provides insight into how they interpret and perform their roles, particularly in supporting patient education, a key component of CKD management. In a context like Cyprus, where the CKD incidence is high, understanding these experiences is critical for enhancing nursing practice and improving patient outcomes.*

2. **What factors do Cypriot nurses identify as affecting their roles in caring for nephrology patients?**

*Identifying the contextual, organizational, and interpersonal factors that influence nursing roles can reveal challenges and opportunities for improving care delivery. This understanding is essential for informing targeted interventions and policy reforms to support nephrology nurses in their practice.*

## 3. Methodology

Because the research questions focus on nurses’ experiences, a qualitative approach was chosen to understand social reality and describe the lived human experiences [14]. Additionally, qualitative research is inherently inductive, with researchers typically seeking to uncover meaning and develop an intuitive understanding of a particular situation [15,16]. The epistemological viewpoint of qualitative research is founded on interpretivism [17], whereas the ontological position of interpretivism is relativism, which holds that reality is not unbiased because everyone perceives it differently [18], and thus, there are multiple perspectives. Consequently, an interpretative phenomenology approach (IPA) was chosen for this study to better understand, in depth, nurses’ experiences concerning a specific situation and what these experiences mean to them [19]. To guide the reporting of our qualitative study, the consolidated criteria framework for reporting qualitative studies (COREQ) was used [20].

In accordance with the underlying principles of IPA [21], the study’s sample was purposive and homogeneous. Permission from the Ministry of Health was required to access the sample, which consisted of nurses working in nephrology care settings across the five state hospitals of Cyprus. Following the approval, a meeting was arranged between the researcher and the administrators of these public hospitals’ nephrology units. During this meeting, an information leaflet outlining the study was provided for distribution to all nurses working in nephrology care settings. The participant information sheet included the researcher’s contact details, enabling any interested individuals to reach out directly with questions or concerns regarding participation in the study. Informed consent was a prerequisite for involvement in the research. Participants were informed of their rights, including the right to withdraw from the interviews at any time without any impact on their employment, through the Participant Information Sheet and the Informed Consent form. Nurses provided their consent by returning the completed consent form via email or fax, using the contact information supplied in the information sheet. More precisely, 16 nurses who had similar experiences and worked in CKD care participated in the current research. The criteria required for nurses participating in the study were as follows:

- Nurses working in nephrology care units.
- Nurses working in public hospitals.
- Nurses speaking the English or Greek language.
- Nurses who have more than one year experience of working in nephrology care units.

The sample consisted of nurses from all district hospitals throughout the entire island of Cyprus. To collect information on the experience of nurses working in nephrology care settings, semi-structured interviews were utilized, as they are the most suitable tactic for gathering data due to the likelihood of stimulating elaboration of interview questions while also keeping a consistent framework across the interviews [22]. The interviews took place at the University of Nicosia or at a location chosen by the participants, whilst the date, as well as the time of the conducted interviews, was determined by their choices. All interviews were transcribed verbatim in the participants’ mother language, Greek, by a trained transcriptionist to ensure the accurate capture of linguistic and cultural expressions, and emotional tone, and then reviewed and verified by the researcher who conducted the interviews to ensure accuracy and capture any contextual details. The transcripts were then translated into English by a professional translator using a meaning-based approach to preserve the participants’ intended meaning and context. To enhance fidelity, the researcher, fluent in both Greek and English and familiar with the cultural setting, reviewed the translations to ensure accuracy and consistency with the original content This study implemented the core features of research ethics, such as consent, anonymity, and confidentiality. The Cyprus National Bioethics Committee issued a letter of approval, and the Ministry of Health gave permission to enter the hospitals to provide the information booklet to potential participants. As this study’s aims were to identify similar experiences of nurses in CKD care, the steps of Smith and Osborn [21] were followed for data analysis, looking for the themes connecting themes, continuing the analysis of other cases, and writing up the results. The first transcript was repeatedly read, with notes and emergent themes (e.g., machine operators) recorded and refined. This process was applied to all cases. Themes were grouped into broader categories (e.g., roles). A second researcher checked coding consistency. A spreadsheet tracked theme occurrence, and only well-supported themes were kept, ensuring rigor. Data saturation was determined through two approaches. First, the Comparative Method for Themes Saturation (CoMeTS) approach [23] was employed to test for thematic saturation in this research. Second, the Hennink et al. [24] approach was used to determine whether the sample size required for thematic saturation was also sufficient for meaning saturation.

### 3.1. Results

This article presents the findings from the 16 interviews with nurses working in nephrology care settings. The interviews were carried out at the five district hospitals of Cyprus as follows: three participants from Hospital 1, four from Hospital 2, one from Hospital 3, five from Hospital 4, and three from Hospital 5. Hemodialysis is provided at all the hospitals: four also provide peritoneal dialysis; three have a nephrology ward where they admit nephrology patients; and only one hospital carries out kidney transplants and accepts patients with autoimmune diseases. In terms of the gender composition of the group, four of the participant nurses were male, and 12 were female. Additionally, their ages ranged from 25 to 55 years, their work experience as a registered nurse ranged from 1 to 31 years, and their work experience as a nephrology nurse ranged from 1 to 29 years (see Table 1).

**Findings**: The interviews were detailed discussions in which six categories emerged for data analysis: they covered several themes with the participants. Specifically, the themes emerged through interpretative phenomenological analysis (IPA), following the methodological guidelines proposed by Smith and Osborn [21], as follows: (1) nurses’ roles in chronic kidney disease (CKD) care; (2) nurses’ preparation to care for CKD patients; (3) organization issues affecting nurses’ efficiency; (4) barriers that prevent them from educating CKD patients; (5) difficult patients; and (6) nurses’ defensive techniques to prevent emotional stress admitted. What follows is a detailed analysis of these categories that emerged from the interviews.

In this article, all the themes, except the first one, will be analyzed since they refer to factors affecting the implementation of nurses’ roles, including patient education.

### 3.2. Nurse Preparation

Despite the various roles participants reported playing, the vast majority felt unprepared to undertake such roles when providing CKD care. Unfortunately, in Cyprus, the only preparation that nurses have for renal nursing is during their undergraduate studies. Therefore, the knowledge they acquire depends on the curriculum of whichever university they attend, which specifies how many hours student nurses need and how in depth they must learn renal nursing. It would be useful in a future stage of this research to explore what the curriculums of nursing at universities in Cyprus include regarding renal nursing. Also, through the interviews, it was identified that only in the last few years have some hospitals started to assign nurses as mentors to newcomers to a ward/unit. However, only two of the participants referred to this new initiative, and remarkably, they were themselves mentors. Additionally, it is important to report that, when nurses are employed in the public sector of Cyprus, they are allocated to the various wards according to hospital needs, regardless of the nurses’ preferences and the preparation they may have had.

One of the findings is that most of the participants highlighted the lack of preparation they had to care for CKD patients, especially when they first started working in the CKD care setting. The following participant, who had only one year of experience, expressed his feeling of unpreparedness as a newcomer in the hemodialysis unit.

*“I think the head nurse here should have trained me…They should have told us two or three things when we first came…not only about the machine and how it works.”*

*(01-M-PA-25)*

He felt that he had not been trained for the work he was doing and that the only training he received was on dialysis machines. Presumably, any additional knowledge and skills are acquired through time and experience.

Participant 03-F-LA-35, who had three years of experience in hemodialysis, stated the following:

*“No, I do not feel ready; do not feel ready…Only yesterday, I heard what the symptoms are of bad haemodialysis, and that our patients have started this thing. And I couldn’t realize this all this time, that is in three years, I mean…I heard this yesterday. Our patients don’t go through good haemodialysis because they have this, that or the other.”*

and

*“Because many times I find myself unprepared, many times…with their fluids, say, what foods should be…Let’s just say at the beginning when I came and [they] told me how to heparinize, how to tie it up. I had many questions.”*

Also, experienced participants observed the following:

*“No, they just said that they had positions in haemodialysis. I knew nothing about haemodialysis, [and] I went, and I just saw machines…It was basically all machines. I thought I would have difficulties to learn how to use them, but okay.”*

*(11-F-NI-45)*

*“I do not think they are prepared. I do not think they are prepared about what they will face here…”*

*(16-F-AM-52)*

It is remarkably interesting what nurse 10-M-LI-35 expressed when asked about his preparation to work in a hemodialysis unit:

*“The first week when I was at home, I heard the “tou-tou [beeping]” of the machine. I was listening to “tou-tou” and I was wondering what is happening now? Until you learn it you do not feel comfortable with the space until you learn to assemble the machine. As new, ok you go with someone, some people. You can go with them for some time to learn, to train in. Let’s say you’re there six/seven mornings this week, you will go with this person out of the staff members to teach you the first week how to assemble the machine and slowly you can enter…”*

He undoubtedly highlighted the psychological intensity of the first days working in the hemodialysis unit, referring to the illusion that he was hearing the sounds of the machines when he was at home.

A participant revealed that newcomers in the hemodialysis unit had no support or any type of training from their colleagues, especially the older ones:

*“Occasionally…by colleagues if I was asking. The workload was great, and they preferred to keep me in the corner…I felt very disadvantaged when I was placed initially in this unit. I felt that I had no support from colleagues, and I felt like I just came from the nursing school…Okay it slowly passed when the older colleagues left…But for a long time, it was like this.”*

*(12-F-LI-34)*

However, participant 14-F-LA-36, who worked at a different hospital, disagreed with the above and expressed that she had received a lot of support from her colleagues. However, her learning and developing knowledge was greatly based on her own efforts:

*“…no education: support yes. I was well received; I cannot complain. They helped by educating me, but I also had personal interest. I wanted to learn. If I hadn’t wanted to, I wouldn’t ask. I wanted to learn because I went to a completely strange place when I was a student for two months. I had never used machines or nephrology or anything like that before. I said I must learn. If I don’t learn so I can see if I like it or not it means…I had no other choice.”*

The lack of preparation was clearly supported by an experienced nurse administrator who had been working for only two years in hemodialysis. She stated that nurses need more than two years of experience to meet the requirements and demands of hemodialysis patient care:

*“They will not perform in all things. They (nurses) need two years’ experience to be able to stand and feel confident. And yet they will not.”*

*(13-F-LI-55)*

The same participant described training sessions provided by medical staff and experienced nurses on nursing topics such as puncturing a fistula, machine issues, and peritoneal dialysis. However, she stressed the difficulties there were in organizing the training sessions:

*“Now we are trying to organize training for some of our staff. They can, for half an hour each time, to learn about nephrology…Ι discussed with the older nurses and decided nurses of next shift to come earlier and attend some sessions about nephrology or pathology etc., when all patients are on the machines, and everything is fixed…We have managed this with great difficulties.”*

*(13-F-LI-55)*

Another nurse administrator, who had worked for 25 years in hemodialysis, clarified the need for preparation. She confirmed that some ad hoc teaching was provided. Nevertheless, at the same time, the participant referred to the limited time available for these teaching opportunities:

*“Ok you need preparation [good preparation]. They need some months to really adapt to the work in haemodialysis. In the beginning, you talk to them about haemodialysis, what it is, how the machines work, what an artificial kidney is. You explain some things, because there is not much time, some things so they can…the only thing they know is that the patient comes, I connect them to the machine, I start it, they stay there for some hours, that’s it we’re done.”*

*(15-F-LA-47)*

Participant 08-F-NI-33 expressed her feelings of unpreparedness to work in a specialized department that also cares for patients who have undergone kidney transplants. Also, she emphasized the gap between theory and practice in relation to a renal setting.

*“I definitely did not feel prepared at all. We went to a large center which dealt with transplants.”*

*“At school we had definitely heard the word ‘transplant’, but we did not go into much detail with this subject, transplant. We needed to learn new things. What is transplantation, what do patients do after the transplant?”*

Another nurse expanded upon that comment and highlighted what was taught during her studies and compared this to the reality of a newly qualified nurse in clinical practice:

*“I was not prepared at all…I didn’t, I didn’t know how to deal with the patient. Although I went to a (nursing) school, yes, I did gain some basic knowledge to build on, but it’s not the same as…having to deal with and being responsible for these (CKD) patients. Things are different in theory and different in practice.”*

*(07-F-NI-33)*

It is clear that, although this participant felt that the nurses’ undergraduate studies inadequately prepared them, she would have preferred to acquire at least the basic knowledge regarding transplants. This was further supported by the following participant:

*“…We don’t have training on this thing (CKD care). That is what did we have in college? Biology, anatomy, pathophysiology…”*

*(03-F-LA-35)*

This participant expressed her feelings of unpreparedness for CKD care because of limited related didactic elements during her undergraduate studies. This was also supported by the following participant, who stated that, even though she had been superficially taught about hemodialysis, she did not undergo any practical training in this specialized area:

*“I mean about dialysis…Yes, we did talk about it, we spoke about dialysis, but we didn’t do…I personally didn’t even go for certain days for some practical training to the dialysis department.”*

*(09-F-AM-33)*

*“No, I did not pass-through the haemodialysis department. I might go through the most amazing department, neurosurgical, where they are specialty departments, from haemodialysis never. We went to the nephrology department, but they didn’t have haemodialysis. I did not have any relation with the department, neither did when I went to nephrology did, they tell me to go to haemodialysis. There was no preparation I think not…”*

*(10-M-LI-35)*

The results highlight nurses’ need and willingness to attend a specialty course in nephrology nursing:

*“I think that is necessary to have a specialty programme in nephrology, which does not exist in Cyprus. In the past, I was searching about it abroad.”*

*(11-F-NI-45)*

*“…Basically, I didn’t do anything more, nothing is offered, we didn’t look for anything more anyway. I didn’t do anything extra like a course, something to follow is not provided, but now I would like something like that.”*

*(14-F-LA-36)*

Furthermore, nurses tried to fill the preparation gap with their personal efforts to develop a knowledge base:

*“But I had no preparation other than the preparation I had myself…Therefore, I started reading about…through websites, through books…”*

*(12-F-LI-34)*

It is evident that undergraduate nursing education in Cyprus fails to prepare nurses to meet the specific needs of patients with kidney problems, which is quite reasonable due to the generality of the education offered through undergraduate courses. However, it raises the question of how adequately the existing undergraduate curriculum in Cyprus prepares nurses to meet the needs of patients with chronic diseases in general. Issues such as the prevention of disease, the promotion of health, and self-care management are vital for all patients with chronic disease, including those with kidney problems. However, it is reasonable that some nurses would not have obtained CKD care experience, knowledge, and specific skills due to the complexity of the renal care settings.

In conclusion, these statements also show that the nurses still felt unprepared despite having extensive experience in the same situations. Instead, they are still gaining basic knowledge, which shows that not only were they unprepared through their undergraduate training for CKD care but they also lack minimal continuing education. Periodic professional development during work would essentially help them achieve satisfactory performance in CKD care settings. Perhaps someone with the status of a mentor would be helpful for new nurses in the nephrology area or a period of adjustment under supervision. This would result in a reduction in anxiety, as most inexperienced nurses show their dissatisfaction with a feeling of unpreparedness.

### 3.3. Organizational Issues

#### 3.3.1. Rotation System

Most of the participants referred to the job rotation system implemented at hospitals in Cyprus. It was apparent that job rotation caused some participants to feel frustration, distress, and a lack of confidence. They attributed these feelings to unsuccessful communication with nursing administration. For example, consider the following:

*“I worked for a year in paediatrics in Nicosia and two years in the military hospital.”*

*(02-M-AM-30)*

*“…the paediatric, pathology, orthopaedic and maternity departments…outpatient clinic”*

*(03-F-LA-35)*

*“I worked in the casualty department in the beginning, and then I did 7 years in Kofinou (primary healthcare centre), 7–8 years. Then, I went to the Agios Georgios elderly home, then 2½ years at the Airport, 4 years…in the First Aid department…4 years in the HIV/AIDS department, at the Gregorio clinic, and now 1 year at the…Nephrology.”*

*(06-M-LA-41)*

*“…The first department I went to anyway was paediatrics; after that they took me for the…the government’s home for the elderly. I went there for some time, then I went to the casualty department…Well, after that, we came to the hospital, we were transferred, and we came to…here. Well ok, I spent about a week at the old one (the old general hospital), close to the casualty department, and then pathology…I went to paediatrics…and then I came here…back to paediatrics…I did the rounds.”*

*(09-F-AM-34)*

Participant 12-F-LI-34 expressed her annoyance with job rotation, clarifying as follows:

*“I once mentioned to one of the head nurses coming from a different field that I had just started my practice in the public service. I said great, now I’m in the nephrology department, tomorrow oncology, then paediatrics. Don’t you think this is something negative for the hospital? Nobody asked me if I wanted to go from paediatrics to renal, and if I like it then great, I might learn how to do the job fast and start learning too, but what if I don’t like it?”*

The perspective of 12-F-LI-34 was interesting because she claimed the rotation system was a form of bullying and punishing nurses who have strong views that may differ from those of the administration:

*“I got an unfavourable transfer…Also, when your views are not expected by some others, you can easily be transferred. I had no arguments with someone, but if you express your views and they are different from others’, you may change ward.”*

She added the following:

*“I had expressed many times in the neonatal unit that it would be good to apply certain interventions to help some children or to utilize specific equipment that I used to have in my unit. Obviously, some in the unit heard and liked my ideas, but some did not like them. Whereas in October I was doing the APLS, which the government paid for, in January I stepped in the unit one afternoon, and the head said: Do you know…(name) that you are transferring in another department? But I didn’t…The head said, someone will move to renal, fine, Zina will move to the renal department.”*

Other participants argued that rotating to interrelated nursing settings boosts knowledge development and the acquisition of certain nursing skills in a specific care field:

*“…Before I was appointed to the hospital, I went to the Paraskevaidio Transplant Centre…Kidney transplants, also nephrectomies…There was a dialysis department…and so I went to the General Hospital, to the dialysis unit for 1½ years. When the transplant centre opened in Nicosia, I was moved to the…nephrology transplant centre, and I have been there ever since.”*

*(07-F-NI-33)*

Furthermore, another participant reported being rotated only in related workplaces. In her whole career, she worked only for a while at a transplant center and then in CKD care settings:

*“I had just finished the school…I had gone to the Paraskevaidio Transplant Centre, where I was hired and since then…I am with kidney patients.”*

*(08-F-NI-33)*

However, the opinion of participant 15-F-LA-47, who is an administrative nurse, provided further evidence that one of the advantages of a job rotation system is to prevent staff burnout:

*“Ok, when you work there though, you understand if you can stay, because indeed sometimes it can be tiring to see the same patients for so many years.”*

Certainly, a rotation system would benefit both nurses and organizations, but it should be available mainly for newly registered nurses and nurses with some experience who may want to develop further in different areas. Moreover, permanent positions must always be available for those who do not wish to move to different departments.

#### 3.3.2. Administrators’ Non-Responsiveness to Nursing Deficiencies

Participants expressed a lack of understanding among the whole organization and nurse administrators in relation to various needs and difficulties that staff face. They discussed the shortage of nurses in hemodialysis units, as well as the shortage of equipment and professional guidelines to meet the demand of the increased number of hemodialysis patients.

*“No, I think not. I think that even if they wanted to, they are not able to understand, because most of the times they see just numbers, even if you are trying to explain to them that we need staff…They don’t understand. We do and we know because we are there living it.”*

*(11-F-NI-45)*

*“I asked for the protocol but, until today there is no protocol…I would like to have more support so that I could offer more support. I would start from the equipment…machines…technicians…Just some reforms are required to gradually increase the number of the machines, because 4–5 years ago when I came to this unit there were 80 patients; now, there are 180. So, some reforms were needed. The staff, I must mention, is still the same number as it was when we had 80 patients…There is not enough staff, and I believe that the administration is not good enough.”*

*(12-F-LI-34)*

Another participant who worked in a small hemodialysis unit commented on the inability of the management to understand the nurses’ feelings of insecurity when there are no very experienced nurses for support because of the rotation system and the absence of a doctor in the afternoons and on weekends.

*“We have raised the issue, sent an official letter about being covered, that we are exposed if something happens, and nothing, no one. Last time we asked for a written statement about who is meant to cover us if the doctor is out.”*

*(09-F-AM-34)*

#### 3.3.3. Inconsistent Expectations from CKD Care Nurses

The data revealed that nurse administrators who are at the level of nurse supervisors and nurses in charge have dissimilar criteria and expectations from nurses working in CKD care settings.

Participant 13-F-LI-55, who is a supervisor with very extensive experience but only two years in hemodialysis, was asked about the criteria for choosing the nurses to work in the hemodialysis unit. Surprisingly, she expected her staff to be willing to accept a schedule that was different from other wards or units and to be quiet as well as energetic.

*“First, in dialysis we have a different time schedule which is not accredited. So, a nurse, to come to the haemodialysis unit, should accept this schedule…I want the nurses who come not to make noise…I want organised people…surely, I don’t want sluggish people.”*

Moreover, the same participant expected only experienced nurses to deal with and manage issues related to dialysis and patient safety. On the other hand, she expected inexperienced nurses simply to keep the equipment and supplies of the unit organized.

*“The nurse came in the morning, have they made their rounds? The patient came, had they have vomiting or had diarrhoea? Had they eaten or drunk a lot? Are they able to evaluate anything that the patient mentions in order to act? I expect all these from the experienced nurses. I do not expect that from the inexperienced. From the middle group, I expect them to have the department organised with oxygen, the crash trolley to be ready.”*

Another nurse administrator with years of experience in CKD care would expect nurses to be understanding of their patients and to always be polite, patient, and persistent.

*“When they are patient and persistent…and polite, very polite…to understand the patients’ problems. To be able to advise and not get angry with what they will hear and not think that that’s the way the patient is and behaves.”*

*(16-F-AM-52)*

One more expectation was that nurses should have broad experience in caring for patients with chronic health conditions.

*“Usually, nurses who have worked in large departments are chosen, surgical department, orthopaedics, pathology—nurses who have learned the basics, and then you bring the nurse to specialisation. First, they gain general knowledge and then specialisation.”*

*(16-F-AM-52)*

*“They are supposed to know that they (patients) have been patients for years. Chronic, that is very important.”*

*(15-F-LA-47)*

Interestingly, nurse supervisors and nurses in charge of the CKD care settings seem not to get involved in the process of selecting nurses to work in these settings. When participant 15-F-LA-47 was asked if she was able to choose nurses who would work in the hemodialysis unit or nephrology ward, she clarified the following:

*“Up to now, no I never have…”*

#### 3.3.4. Opportunities for Continued Professional Development

It is well known that nephrology nurses, as healthcare professionals, always need to update their knowledge and skills, and continued professional development (CPD) is important in CKD settings. In contrast, the participants in this research highlighted the difficulty of attending a nephrology nursing conference or seminar, the unavailability of or having no access to scientific journals at work, and the absence of a nephrology nursing course.

For example, a participant with three years of experience in a hemodialysis unit stressed that the nurse administrators had negative attitudes towards conferences and seminars and did not encourage nurses to take part and attend. Also, the participant strongly expressed her disappointment and annoyance using idiomatic language.

*“There is another problem in the unit, let’s say, where the staff cannot go anywhere…‘You will not go to the conference in Limassol’. You will pull your hair out, not one person attended the conference from the nephrology department, and the reason (of the administration) was not enough staff. I will go crazy!”*

*(03-F-LA-35)*

One more participant from the same hospital also supported the limited number of nurses attending conferences.

*“Unfortunately, not all of us can go. This year these five nurses go, the next year these five…”*

*(14-F-LA-36)*

Another very experienced nurse in CKD care suggested that the nurse administrators should facilitate the attendance of conferences to enhance nephrology nurses’ capability to educate their patients.

*“Sending you to different conferences…this could help…”*

*(08-F-NI-33)*

Even a nurse supervisor in hemodialysis said that nurses could attend conferences if they wanted but confirmed the difficulty of allowing nurses to attend conferences because of the heavy workload.

*“I did not allow many nurses to attend. I wanted to let them attend but they realised themselves that they could not go. Unfortunately, in the theatre it was very different. There were conferences only on Saturdays when only the team for urgent cases was on shift. There were many nurses attending. Here it is difficult.”*

*(13-F-LI-55)*

It is, however, worth mentioning that there was some doubt about the quality of the conferences, as they do not provide the expected learning outcomes.

*“I think they are poor because they don’t…If they cannot produce an educational programme, say within a short period of time in two days, to remind us of the basics, to teach us the basics…We could listen to what new research says.”*

*(04-F-LI-27)*

In addition to encountering obstacles to attending conferences on nephrology nursing, CKD nurses did not have access to any scientific journal through their work organization.

*“I have not seen anything [scientific journal] related to nephrology nursing.”*

*(03-F-LA-35)*

Many participants, independent of the extent of their experience, pointed out the importance of a special course in nephrology nursing and their willingness to attend it if there was such a course.

*“It is an area that needs specialisation. I would prefer to go through a course…Without specialising, I do not think that you could succeed in such a field.”*

*(03-F-LA-35)*

*“It would be good if we had specific training…a specialisation.”*

*05-F-LI-45*

Despite participants’ wish to take a course to specialize in CKD, the nursing administration does not seem to share the same perspective. Participant 12-F-LI-34 clarified as follows:

*“In our field, you get promoted based on years of experience and service in the department. At the hospital, qualifications do not matter; the only thing that matters is whether you have graduated from nursing school…”*

The same participant revealed that, when she expressed to her administrators the need for a specialization course, they reacted negatively by criticizing her suggestions and giving no choice in a provocative and unprofessional manner.

*“They didn’t like it at all…They even said what are these ideas you bring. That’s what the hospital offers, either you like it or not…”*

The nephrology nurse administrators’ attitude to the provision of a specialization course was confirmed by an experienced participant who revealed there was no discussion about such a course, although he would be ready to attend it:

*“I mean, I know for example that for the intensive care unit they may send you on an intensive care course. I have never heard about the dialysis course…I would like to do it, I like the…dialysis.”*

*(06-M-LA-41)*

It is clear from the above that CKD nurses are eager to improve and extend their knowledge and skills by attending more conferences, having access to scientific journals, and completing a specialization course in nephrology nursing. Administrators should perceive the willingness and needs of their staff and try to meet them by providing related opportunities.

#### 3.3.5. Nursing Autonomy

Very few participants referred to having autonomy in working and delivering care to CKD patients.

A young nurse expressed his disappointment because of a lack of cooperation and support from older colleagues, as well as restrictions from doctors who do not actually allow him to undertake any initiatives.

*“What I wanted to say before is that there is no cooperation here. That is, if I take the initiative. Because, I have heard many times before “who are you”, that this is something that prevents nurses from wanting to cooperate…There are some here who think they have power. I apologise for speaking in such a way.”*

*(01-M-PA-25)*

Another participant with little experience in CKD care confirmed that doctors do not allow nurses to implement certain interventions, such as patient education.

*“Yes, it’s…let’s say that thing…the doctors, doctors…you stay out of it, just do your part, standard care…We have reached a worsening situation, just a parenthesis, we have subclavian, right? And if one seam is cut, we must call the physician. Until recently the nurses also had to go. Now they have started (to say) if the wound is infected, you do not know how to sew, and the doctor must come.”*

*(03-F-LA-35)*

However, a nurse from a different hospital disagreed with the previous participant by saying that the interprofessional relationship between nurses and doctors has improved.

*“Okay, the way doctors see us has changed, it is not as before. They see us as professionals, and we earned this.”*

*(05-F-LI-45)*

It is apparent that team spirit boosts nurses’ feeling of autonomy when they are expecting support from and collaboration with coworkers and medical staff.

### 3.4. Barriers to Patient Education

As has already been mentioned, patient education is a fundamental part of patient care, and it is one of the important roles of nurses. However, poor education is the most common source of patient complaints in the healthcare sector [25]. Since one of the findings of this analysis was the absence of patient education in some cases, participants were asked to report factors that prevent them from carrying out their role as patient educators. Most of them referred to inadequate guidance or support from administration, staff shortages, a lack of available time, limited knowledge, and nurse–doctor boundaries.

#### 3.4.1. Inadequate Administration Guidance

There was a conviction among most of the participants that they do not educate their patients due to the lack of guidance or support from nursing management. It was also expressed that the ward’s head nurse, who is perceived as one of the most important persons of the administration, has the authority to support or guide them in educating patients. Importantly, this was the view of nurses working in different hospitals.

When participant nurse 03-F-LA-34 was asked whether she felt that the administration provided any help and support to execute patient educational activities, she just replied as follows:

*“Nothing.”*

Certainly, her absolute and monolectic answer emphasizes the lack of support from the administration. The following comment by participant 02-M-AM-30 charged the responsibility for implementing patient education activities to administration and stressed that the head nurse must give nurses permission to educate patients.

*“The administration, let’s say, or the head nurse will give us guidelines that we should educate.”*

This was further supported by participant 04-F-LI-27, who expressed that she expected that the administration would encourage and even demand patient education:

*“To give incentives, let’s say. To motivate us…”*

*“If the heads of departments encourage you, if they tell you it is part of your job, if they keep an eye on you.”*

The need for the administrators to incentivize nurses to educate patients was also confirmed by participant 10-M-LI-35, who has eight years of experience in a hemodialysis unit. Even when he was asked whether he feels ready to carry out patient education, he pointed to the existence of a policy that would mandate patient educational activities.

“If the administration comes and tells me that they want teaching to be a part of our daily tasks, I will be planning, organizing, etc…It is good to have the theoretical (basis), to have the knowledge; it’s a very good thing. But, ok…it is a matter of policy of the unit.”

Additionally, participant 07-F-NI-33, who has many years of experience in the field, also supported the notion that there is no guidance from the administration regarding patient education:

*“Teaching? Do you mean about teaching the…Let me tell you something, I think that…many times, they overlook it…The same way that everyone else does. Because…they see all the other serious things that need doing on the ward, and they forget about the patients, what the patients themselves need to learn.”*

That statement shows that the administration does not encourage or guide nursing staff to educate patients because they must carry out other tasks. That was previously observed that nurses focus on completing tasks instead of holistically caring for patients. Additionally, it was supported that the administration failed to plan well to incorporate patient education into daily nursing practice.

*“There is no good programming in various fields to give the opportunity to the staff to implement their educational role as it should be. There is no administration to impose a programme that includes patient education so that patients would be informed.”*

*(12-F-LI-34)*

It was additionally remarked that there is no written guide on various topics for patient education either for nurses or for patients.

*“…No, there is nothing written down. Not as far as I know…for fistulas for example, I don’t think there are any instructions for the patient. We just have instructions on how to puncture the fistula. Not instructions, not a protocol. We don’t have something for the patient. We do it personally.” *

*(14-F-LA-36)*

It is obvious that the lack of administrative support and guidance is an inhibiting factor for nurses in educating patients, and it can be strongly correlated with the absence of encouragement for nurses to perform educational activities.

#### 3.4.2. Shortage of Staff and Lack of Time

One of the most reported barriers to patient education was the shortage of staff and the consequent lack of time to complete all of the activities. Τhis situation is further burdened due to the various tasks and responsibilities nurses have, many of which do not even belong to nursing practice. The shortage of staff was highlighted by a well-versed nurse (10-M-LI-35) in the CKD care field who clarified the following:

*“…We are dealing with stifling situations. We are in stifling situations…reduced numbers of nurses do more beds now…*

*“We have no time…Yes, this is the reason we have changed time schedules in haemodialysis unit…many patients. We work at different hours than any other departments of Cyprus…The issue is on the reduced number of nurses.”*

Also, experienced participant 08-F-NI-33 referred clearly to the shortage of nursing staff in Cyprus and repeatedly suggested that more nurses need to be employed.

*“There is no way that we have the time in a ward where there are 16 patients. The staff may be reduced daily due to sick leave, and due to the fact, that…they are not hiring people now…”*

*“Hiring more staff would be a way, so that there is a better distribution of work, a different distribution of work, so that you can dedicate more time to the patient…”*

*The nurse administrator 13-F-LI-55 confirmed the above, referring to her difficulties in dealing with the continuously falling number of nurses.*

*“…We try to prepare them (nurses) not to take vacation during the summer if the situation continues like this. Of the 38 nurses, [some] will be on maternity and sick leave…and will return in six months…They are not replaced, nor when they are on maternity. Last year I had three staff members on maternity leave…plus a sick leave due to a surgery…I had 34 nurses left. It was great pressure.”*

The problem of the limited number of nurses was also emphasized by another administrator, a senior nurse officer, who mentioned her regular involvement in the work routine of her hemodialysis unit.

*“In general, my job is more administrative, but the supervisor nurse working in haemodialysis is not only an administrator but has to work with the patients like the rest of the nurses. Because of the lack of staff, it is necessary to get in the department and work with everybody else. Maybe sometimes we work even more than others.”*

*(11-F-NI-45)*

On top of that, the multitasking environment of the participants leads to time pressures that do not allow the implementation of certain nursing actions, including patient educational activities.

*“We carry out hundreds of tasks that are not nursing, dealing with everything else apart from…How much paperwork…We right in messages, about the patients, into the computer system, you brought such and such form, you didn’t bring in such and such form. What…a hundred thousand things.”*

*(03-F-LA-35)*

*“The nephrology ward is a mixed ward where various things happen; you don’t have the time to do so [patient education]. It would be very good if we were able to do it, like when we have a transplant patient.”*

Moreover,

*“Lack of time…yes, workload. Many patients, many demands…and just so you know the chronic patients are much more demanding than the patients who will come in for a procedure and leave. They may ring the bell about 20 times each on every shift, and quite often without a serious reason. But you must be there to respond. So, the things that you want to do, you can’t always do.”*

*(07-F-NI-33)*

*“…There is a lot of work to be done, as I mentioned, we have dialysis, peritoneal patients, transplants. There are many things in the department. We don’t have time left to deal with any patient one on one to teach them.”*

*(08-F-NI-33)*

The lack of patient education was justified by participant 12-F-LI-34, who was asked whether nurses achieve their roles including patient education, and she simply answered as follows:

*“Not really…due to heavy workload and lack of time…”*

Similarly, the experienced nurse 14-F-LA-36 explained the reasons for not educating patients:

*“…It’s the workload…and the lack of staff many times. There isn’t enough staff. I mean, for each nurse there are four patients. We can’t just start chatting…”*

On the contrary, one participant who works in a small dialysis unit in a provincial hospital with only six dialysis machines claimed that nurses there have time to educate patients, as they have only a small number of dialysis patients. Specifically, this participant reported the following:

*“The truth is that we do have some time. There are fewer patients in our unit.”*

*(02-M-AM-30)*

Of course, this opinion is the exception, as this specific participant was working in a satellite hemodialysis unit that has only five patients daily. When the patients face any problems, they are referred to another larger hospital.

Concluding the above, nurses confirmed the shortage of staff and lack of time and clearly considered them obstacles to patient education. They felt that their routine job is very demanding and does not allow them to conduct activities such as educating a patient.

#### 3.4.3. Limited Knowledge

The participants considered having limited knowledge to educate nephrology patients another preventative factor for patient education. For instance, consider the following:

*“I lack the knowledge. Well, I have power, but I am missing knowledge.”*

*(03-F-LA-35)*

*“Sometimes we may not know our subject very well. We ourselves may not know how to…how to teach the patient…The correct way to teach…”*

*(07-F-NI-33)*

*“It is the lack of knowledge, lack of time. The two major things I can think of now.”*

*(04-F-LI-27)*

Participant 14-F-LA-36, who is very experienced, confirmed all of the above:

*“But on more specialised issues I can’t say that I have enough knowledge to explain something…But there are things on the machine I don’t know. Some things I want to learn. But unfortunately, we haven’t…”*

When a nurse administrator with 25 years of experience in CKD care was asked to rank the knowledge of her nurses in the CKD care settings, she simply answered as follows:

*“Inadequate.”*

*(15-F-LA-47)*

Participants identified nurse education as the source of the extra knowledge needed.

*“Education…because experience is not enough, say in relation to the time the experience we gain is not enough…to be able to properly educate a patient who required haemodialysis.”*

*(04-F-LI-27)*

The interesting part of this statement is that the participant identified that experience alone is insufficient and extra education is required.

Additionally, most of the participants understood that their lack of knowledge left them unprepared to educate CKD patients. Participant 08-F-NI-33 supported the notion that nurses who were new to working in the nephrology field were unprepared to educate patients at the beginning. She clarified that, only after gaining experience, did she know what information was needed and how to educate her patients:

*“Well…I didn’t feel prepared at all. I can’t say that I felt prepared because I gained experience through work, and that is where I understood what it is I should advise on and how to…teach the patients.”*

Participant 07-F-NI-33 reported that, when nurses completed their studies, they might be prepared to educate patients on general matters. But, as they are not being adequately prepared for nephrology patients, they have to study on their own to learn more:

*“Well, I wasn’t ready. I couldn’t do it to the degree…And I can tell you that my part in teaching about medication from the time that…I was in my first or second year…I was prepared to do it…I wasn’t this way with all subjects though. It’s just that this subject appeared to me. I made an effort, studied, educated myself, I opened books, I looked things up online…I didn’t stay where I was when I finished nursing school.”*

The participant was motivated by the new subject of her work and tried to meet the requirements through personal initiative and effort to improve her knowledge.

Also, participants revealed that, during their undergraduate studies, patient education was only mentioned but not emphasized.

*“At the school…This was always…psychological support. Teaching…it may have been mentioned, but we didn’t give that much emphasis to the matter.”*

*(08-F-NI-33)*

It is of great interest that nurses are not prepared during their undergraduate studies to educate patients, although the patient education role is a mandatory responsibility of the professional nurse. It is really important here to highlight that nurses are unprepared to educate CKD patients due to the lack of continuing education, which could be because there are no administrative strategies for confronting this issue.

When participant 05-F-LI-45 was asked about nurses’ preparedness to educate patients, she stated the following:

*“In school, yes, what about afterwards, however? Before I came to the department of haemodialysis, there could have been a course, two to three months, so we could integrate better…”*

*(05-F-LI-45)*

Likewise, participant 12-F-LI-34, when discussing the expected outcomes of a course in nephrology nursing, made clear the following:

*“Knowledge not just practice…deeper knowledge. The ability to help the person, I must deal with all aspects, rather than just entering a ward and not being familiar with the subject”*

It is obvious that nurses feel that their knowledge is too limited to undertake the role of educator for nephrology patients. This situation is probably due to not having enough support and the fact that the department does not follow a strategy that would allow nurses to smoothly integrate into the field of nephrology.

#### 3.4.4. Nurse–Doctor Boundaries

Some participants identified patient education as belonging to doctors’ duties. The interdisciplinary boundary between nurses and doctors obviously acts as a barrier for nurses’ involvement in patient educational activities.

A participant not only reported that doctors have the duty to educate but also implied that doctors constrain nurses from patient education and tell them to remain within the limits of physical care.

*“Yes, it’s…let’s say that thing…the doctors, doctors…you stay out of it, just do your part, standard care.”*

*(03-F-LA-35)*

The same participant particularly recognized that, even if nurses have the knowledge to educate patients, they do not have the right to do so because their professional responsibilities end where doctors’ responsibilities begin:

*“I may have the knowledge, but the constraints due to some other conditions do not allow us to express, to talk about this thing. Our jurisdiction stops here, and there begins the jurisdiction of the physician. We have nothing to say.”*

*(03-F-LA-35)*

It is obvious that this participant is worried about crossing the doctor–nurse boundary. She indicated that there is no clarity between nurses’ and doctors’ responsibilities. Also, participants recognized doctors’ precedence in providing advice and information to patients and saw their role as just supplementary.

*“Normally, the patient should go through the doctor first for advice. Not what to be careful of, of course, we can tell them what to take care of, but ok.”*

*(01-M-PA-25)*

This was also expressed by a participant with only one year of experience in CKD care, who supported the notion that doctors inform patients about their fluid restrictions and diet, and nurses rather encourage them to be careful in general.

*“The doctors (inform the patents)…and we also inform them that they must be careful.”*

*(06-M-LA-41)*

Additionally, when the participants were asked who informs and teaches patients at the beginning, one participant replied as follows:

*“…I doctor”*

*(12-F-LI-34)*

It is remarkable that nurses either do not recognize patient education as their role or are unwilling or even frightened to cross any boundaries, even though patient education has long been considered a mandatory and independent nursing role that meaningfully influences patients’ health and quality of lives.

### 3.5. Difficult Patients

The increasing prevalence of chronic disease worldwide is a challenge for nursing, as it evokes concerns about several issues, such as patients’ quality of life, healthcare costs, and a workforce that is adequate to meet the rising demands for healthcare services. Having chronically ill persons become more involved in their healthcare by participating in self-management programs is a particularly necessary step. However, despite the best intentions and efforts on the part of the nurses and other healthcare professionals, the expected outcomes may not be attainable if the patients are non-compliant. This shortfall might have serious and harmful effects for disease management. This part of the chapter provides a detailed discussion on the selective category of “difficult patients” and how they behave and react in particular.

Many participants expressed the tendency of numerous CKD patients to abandon themselves, leading to devastating outcomes for their disease progress and health conditions in general. Participants highlighted patients’ anger, as well as their non-compliance with required regimens, due to their negativity in following health professionals’ instructions. Participants’ views and experiences revealed the demanding aspect of many chronic CKD patients and their refusal to accept their illness, which leads them to resignation. While the transcripts were coded, certain themes were identified and gathered into more substantive categories. Each category was analyzed, and several of these were brought together into the category of “difficult patients”. Each of the themes under this topic is discussed below.

#### 3.5.1. Self-Neglect

Many participants stated that there are patients who have let themselves go and were responsible for reaching the point of end-stage renal disease and needing a transplant.

*“Might have diabetes, does not take care, this caused renal failure. Has blood pressure, not careful, yes, okay, if someone’s stung by a scorpion, it is not his fault…there are also those incidents. But most of [their problems] are due to neglecting themselves and they really need some advice.”*

*(10-M-LI-35)*

Blaming patients who have a chronic condition such as diabetes mellitus, hypertension, or any other condition that led them to have chronic kidney failure was evident. Many participants supported the notion that these patients have their current health conditions because of their own negligence in managing their diabetes mellitus or controlling their blood pressure or any other health problem. For example, the same participant stated the following:

*“If someone was diabetic, say, most diabetics have hypertension, most are dialysis cases. Usually they were not taking care, so they ended up on haemodialysis.”*

*(10-M-LI-35)*

Another two participants shared the same opinion. They maintained that patients with a chronic disease, mainly diabetes mellitus but also hypertension and hereditary diseases such as polycystic kidney disease, give up and are non-compliant with their care and treatment recommendations.

*“The first reason is diabetes, I think. There are many cases that are much neglected; they do not follow the necessary analyses and procedures over time to keep an eye on their health. We have a lot of diabetes cases in Cyprus, hypertension, hereditary diseases…”*

*(11-F-NI-45)*

*Interviewee: “You know? To end up here means that you gave up on yourself.”*

*Interviewer: “They gave up on themselves?”*

*Interviewee: “They may notice the symptoms and go to the doctor on time. But if they ignore them, and they say, ‘I’ll go next year’, they ignore it and they end up here. If they cared more when they were younger…”*

*(16-F-AM-52)*

According to the participants, these patients could not accept their illness, and consequently, they could not cope with their health problem, the treatment demands, the daily efforts, and the outcomes of their illness in relation to their quality of life.

When participant 14-F-LA-36 was asked to clarify why she mentioned that some patients were not very cooperative, she gave as an example the case of an educated patient who, despite his knowledge and awareness of his health situation, had not complied with food and fluid restrictions.

*Interviewee: “A certain patient is an educated man. Now he is 62 years old, he studies the machine, he knows many things, he has a lot more health problems too and he says that he will enjoy the rest of his life. He doesn’t want to lose some things.”*

*Interviewer: “He doesn’t follow his diet…his water restrictions?”*

*Interviewee: “Yes, yes.”*

Participant 01-M-PA-25 referred to another similar case where the patient seemed to be indifferent to a serious problem that can progress into a life-threatening situation.

*“Yes, because I see some, those come with infections all the time. We have one girl who pays no attention at all to her infections…She has subclavian, and she takes antibiotics vancomycin and gentamycin. Yes, now it has been three to four months that we prescribed her with Apotel (paracetamol) all the time to prevent her fevers.”*

The fact that some patients are unresponsive to treatment can also be seen by their unwillingness to even communicate with nursing staff. They will not share anything if they are not asked to, or they want to leave as soon as they finish their hemodialysis session.

*“Some people won’t tell you anything if you don’t ask them…If you ask them, they will tell you.”*

*(15-F-LA-47)*

*“A goodbye and they leave. Because I think this is also the aim of the patients themselves, to finish and leave quickly. The patients don’t even sit for five to ten minutes after their haemodialysis, which they have to. They don’t sit and they leave immediately.”*

*(01-M-PA-25)*

In conclusion, the diagnosis of any chronic illness, including chronic kidney failure, confronts individuals with a collection of tasks that they must physically and psychologically adjust to. It is more than necessary for the patients to accept the disease, acquire new skills, and change their daily routines to manage the symptoms of the illness or cope with the demands of treatment. The individuals’ views about their illness determine how they respond to their illness on a behavioral and emotional level. Therefore, it is important for healthcare providers to explore patients’ personal beliefs about the seriousness of their illness, their ability for self-control, and the impact of the illness and treatment on their daily life.

#### 3.5.2. Negative Attitude to Receiving Instructions and Learning

The emerging data from the interviews stresses that many patients are unwilling to accept advice, instructions, and information from nurses. The participants reported that patients claim that they are aware of everything and even make provocative statements, such as that they do not drink water at all.

*“There are many times when the patient may be negative against you and say ‘I know these things; I don’t need you to tell me; I know these things, I am the patient; and I am the best doctor for myself. And I don’t need you to tell me these things.’”*

*(08-F-NI-33)*

According to this participant’s perspective, patients argue that they know everything and they do not need any further information or instructions. Most probably, these patients perceive themselves as unable to control their illness, and they struggle to cope with the fact of being dependent. Similar aspects were shared by another participant who highlighted patients’ negativity about following instructions by stating the following:

*“…So it wasn’t that she could not understand, it’s just that she was a very stubborn woman, how else can I say it, and she didn’t want, she would not accept to talk about her fluids, her diet etc…or about her fluids, I explained to her, and she tells me ‘I don’t drink water’.”*

*(09-F-AM-34)*

The participant’s characterization of the “*stubborn woman*” and the patient’s challenging words, “*I don’t drink water*”, indicated a strong unwillingness to listen and follow detailed information or orders. The same participant described another patient who denied and doubted his doctor’s instructions to start insulin therapy and several patients who generally refuse to care for themselves.

*“Like today, one gentleman we mentioned…for example, the doctor told him to go on insulin. You need to go next door to the diabetes clinic, for them to examine you for insulin. And he said, ‘But not such food. My wife makes it this way and that way, and I don’t know what else, you understand’. He is in denial…you understand? And we are talking about insulin, not about dialysis…”*

*(09-F-AM-34)*

*“But I believe that this is also up to the patients themselves. I mean, it is what I mentioned earlier. There are patients who are accepting and really do try based on what you tell them, and this helps them. There are patients who are always in denial and don’t help themselves…”*

*(09-F-AM-34)*

Negative patients seem unwilling to change their attitude even when health professionals make special efforts to help them, but they remain indifferent and stubborn.

*“Most of them come with high potassium levels, so…They don’t follow their diet. I have repeatedly brought a dietician. He saw them, told them, gave them their diets in writing, and I just reached the conclusion that they, I don’t know…they are weary and exhausted patients…”*

*(16-F-AM-52)*

Furthermore, the same participant pointed out nurses’ endeavors to help patients, but at the same time, she charged patients with their own responsibility to convert their negativity into acceptance and compliance. Furthermore, she specified that the patients’ negativity is due to their lack of concern and fear.

*“The nurses interact with the patients. But they [patients] must try themselves; some of them are very negative and so scared that they don’t want to know…There are also the indifferent ones; there are the frightened ones, the cowards…I don’t want to know—tell my wife; I better not know…”*

*(16-F-AM-52)*

Interestingly, participants 09-F-AM-34 and 16-F-AM-52 work in a very small hemodialysis unit where, according to emerged data, nurses have the time to communicate enough and establish strong interpersonal relationships with patients.

Participants also surmised that the patients’ negativity is related to the Cypriot character and culture.

*“We…Cypriots, have no discipline. The patients…yes, yes. They do not follow instructions, do whatever they like, and it is very difficult…And you should train not only the patient but also the people around him.”*

*(05-F-LI-45)*

The topic of patients’ unwillingness to learn was raised by participants who argued that it was part of their character, as was the resulting responsibility for managing their own health issues. The unwillingness and indifference of these patients to learn was underlined by an experienced nurse, who shared this observation of patients’ attitudes in the hemodialysis unit where he works:

*“…The patients say, ‘Put me on quickly and I need to leave, I have a problem.’”*

*(10-M-LI-35)*

Respondent 05-F-LI-45, who was very experienced in CKD patient care, highlighted that patients and their families were unwilling to be educated, and she argued that this is due to the culture of Cypriots, as they tend to be afraid of responsibility.

*“They [patients] show indifference to their education…I think it’s our culture in Cyprus…It has happened to me not only for the patient but also, in his environment, for his family to be negative to education. They did not want to learn, did not want to…They did not want the responsibility.”*

The patients’ fear of learning and knowing about their health problem, treatment, and their own participation in the management of their disease was highlighted by participant 16-F-AM-52, who stated the following:

*“…Some of them [patients] are very negative and so scared that they don’t want to know.”*

Another participant clarified that only young patients are willing to ask questions and learn about their health conditions and treatment.

*“…Mostly the young people ask questions.”*

*(12-F-LI-34)*

In conclusion, the data that emerged revealed that some patients refuse to listen to their health professionals and follow instructions that they receive from their doctors and nurses. This is possibly because patients feel disappointed and helpless, as they have lost their autonomy, resulting in them developing passive coping strategies such as denial and avoidance.

#### 3.5.3. Denying Reality

The data indicated that patients refuse to deal with their illness and all relevant restrictions imposed by the illness and its therapy. They also tend to withdraw into themselves.

A very experienced nurse in the field of CKD care clarified the following:

*“There are patients who do not accept their illness and want to withdraw…they withdraw into themselves…despite the fact that now most patients are already informed about these things…err, I mean that with their admittance to the ward and their diagnosis from the doctor, err…most of them know about what they should do;…they know what their illness is and what they need to do…”*

*(08-F-NI-33)*

In addition, another nurse administrator with 28 years of experience in nephrology care confirmed the above by saying:

*“Most of them don’t want to believe it; ‘I don’t have high blood sugar.’ ‘You are diabetic.’ ‘No, I don’t have high blood sugar.’ The time comes when they must take insulin. ‘No, I don’t have high blood sugar,’ they insist. Denial…”*

*(16-F-AM-52)*

Although they are aware of the situation, it seems that patients reject a frightening diagnosis and its consequences from the fears that emerge from the whole situation.

#### 3.5.4. Being Angry

The data revealed that anger and frustration are common among patients with kidney disorders because of the outcomes of their disease and therapy.

Participant 16-F-AM-52, who has spent her whole career of 20 years dealing with and caring for CKD patients, strongly expressed the anger and aggressiveness that come from such patients. Also, she referred to the psychological and violent consequences for nurses, but she understood.

*“…Most of them are angry even with God…They are here all the time; they are negative and angry. Normally, they should see a psychologist every week. But they wouldn’t accept that. It’s truly difficult and wears you down…”*

And she continued:

*“I know that the patients are angry, that they might insult you or say something mean. Listen, our patients’ behaviour is expected. I expect this type of behaviour. First, they are angry because they are not getting better for example. They might be angry with the nurses, the nephrologists, and with everyone because they have this condition. I understand that…One patient attacked me the other day, but I didn’t pay much attention.”*

A nurse administrator confirmed the aggressive behavior of some patients:

*“When I talk with the patients, some nurses tell me that this patient was upset…”*

*(13-F-LI-55)*

There was one interesting comment from a hemodialysis nurse who blamed aggressive patient behavior on the nurse administration since she believed they actually allow the patients to be undisciplined:

*“It is a matter of lack of support from the administration. Because they tell us to do whatever they tell you so that they don’t shout.”*

*(12-F-LI-34)*

Recognizing patient anger is necessary for ensuring nurses’ and patients’ safety.

### 3.6. Nurses’ Defense Techniques

Nurses who deal with difficult and stressful situations tend to react defensively in their effort to prevent emotional exhaustion. According to the findings of this study, nurses’ defensive reactions consist of keeping a distance and showing disinterest.

Participants revealed that some nurses seem to be consciously indifferent to either colleagues or patients when they want to escape from situations that could possibly cause them psychological stress. An experienced nurse expressed her disappointment in some colleagues who did not support other nurses in the unit, as is expected. Also, she commented that their indifference had negative outcomes for the whole nursing team performance.

*“…With colleagues, uh…, okay with some we have very good cooperation with others. As I told you, they are indifferent and that is where you become angry.”*

*(05-F-LI-45)*

And she added the following:

*“Because you see that one day the team can fly and the next day when you may have one who is indifferent, no…It affects everyone.”*

*(05-F-LI-45)*

A nurse with 15 years of experience in CKD care disclosed that several nurses focus on the safety of patients during the dialysis session, but they do not care about what will happen to the patient afterwards.

*“I think that in every shift they want to feel safe during the haemodialysis session. Now, when they finish their treatment is their own matter…”*

*(11-F-NI-45)*

The indifference of some staff was also confirmed by two participants who argued that there are nurses still giving their best and supporting their patients, whereas other nurses keep a distance and show disinterest in their effort to protect themselves.

*“Some of us are strong and can handle it and fight and are still close to the patient, some of us cannot handle it. And by trying to protect themselves, they hide behind the mask of indifference, behind the mask of the typical professional.”*

*(12-F-LI-34)*

*“There are staff that won’t bother. They just see the patient and go. They don’t…they just do the necessary things.”*

*(14-F-LA-36)*

There is no doubt that nurses’ indifference can lead to poor patient care, and nursing management should identify the sources of it and support nurses to overcome all the triggers of their disinterest.

Participants revealed some distancing strategies that nurses employ during their practice because of the fear of being unable to handle their own emotions. It was clearly expressed that these nurses perform the necessary interventions for the patients’ hemodialysis session but nothing more than that. They connect the patients to the machines and then keep an emotional distance.

*“Yes, they go in the ward, do their job, insert two needles, insert a catheter, do you need anything Mr. Andrea? Yes or no and depending on that they proceed and distance themselves.”*

*(12-F-LI-34)*

The very experienced participant 11-F-NI-45 confirmed that nurses keep a distance from their patients to prevent developing a closer interpersonal relationship that could lead to negative outcomes for them.

*“You are going to hear him (patient), but it is what I mentioned earlier. You must be a little reserved because then they will be entirely dependent on you, and this negatively affects your own psychology. Then something could happen against you.”*

Keeping a distance, which colloquially is “being a cold fish”, seemed to be a defensive technique. However, ideally, nurses should be capable of separating themselves from their work and protecting themselves from becoming emotionally burdened.

## 4. Discussion

### 4.1. Nurse Preparation

Another outcome of this research is that the majority of participants emphasized that they were not trained to care for CKD patients when they first began working in a CKD care setting. In terms of the amount of information and abilities needed in such a specialized area, participants felt that basic undergraduate training was not enough because of the minimal theoretical courses and practical learning. Nevertheless, it is reasonable that some nurses would not have CKD care experience, knowledge, and specific skills due to the complex nature of these renal care settings. However, according to Benner [26], nurses should feel that they have mastered the ability to handle contingencies in the clinical setting after three years of experience. In contrast, the participants of this study demonstrated their feelings of being unprepared despite their years of experience in similar situations. Instead, they claimed they were still gaining basic knowledge, which shows that not only was the undergraduate training inadequate for CKD care but continuing education is lacking or minimal.

The need for nephrology nurses to receive specialized training in CKD care has been stressed in the literature. Nobahar and Tamadon [27] concluded in their qualitative study about the barriers to and facilitators of care for hemodialysis patients that nurses require specialized training to use dialysis equipment and manage problems arising from hemodialysis. Additionally, the significance of enhancing nursing staff training was highlighted in other studies. Shahdadi and Rahnama [7] argued that a higher nursing education level was among the main contributing elements to the hemodialysis care provided. Providing dialysis education programs and upgrading the educational level of nurses have been highlighted as two key facilitators of efficient, optimal, and high-standard care. Tuyisenge et al. [28] also underlined that the needs of so many patients outweigh the time and human resources available and that there is a compelling need to increase the size of the nursing staff and the level of training. These findings reinforced the outcomes of an older study that highlighted the lack of continuing education for nursing staff [29].

Additionally, it was found through the interviews that only two hospitals have recently started assigning certain nurses to mentor new nurses to the ward/unit. However, it is notable that only two of the participants—who were also mentors—referred to this new endeavor. On the contrary, other participants revealed that newcomers to the hemodialysis units had no support or any type of training from their colleagues, especially the older ones. Similarly, others believed that there should be somebody in the unit when they begin working there to give them general training. The absence of an appropriate induction approach for new staff, including newly qualified nurses, was expressed in an earlier study [29].

Shahdadi and Rahnama [7] found that caring for hemodialysis patients is linked to nurses experiencing negative personal effects (physical damages), mental effects (misconduct, bad temper), feelings of burnout, obsessions with one’s health, feelings of anxiety and depression, a tendency to quit the department, and negative family effects (neglecting children, being unable to meet spouse’s needs, being unable to perform housekeeping duties, and interfering with family matters). Myers et al. [30] supported the notion that a single instructor was favored by the majority of newly licensed nurses. Several newly qualified nurses (RNs) mentioned the need for instructor feedback to reassure them that they were working safely. The stress level of newly licensed nurses increased significantly when they did not receive feedback, and they tended to refrain from asking their instructor questions. This was strengthened by the findings of this research that one newcomer suffered psychological tension that led him to believe he was hearing the noises from the dialysis machine while he was at home.

### 4.2. Organizational Issues

The standard of healthcare is one of the most crucial elements that affects how individuals perceive their quality of life [31]. The struggle to provide healthcare that is both economically viable and of excellent quality is at the same time the top issue for healthcare organizations worldwide and has led healthcare and health systems everywhere to make extensive adjustments [32]. Organizational issues are considered to be the foundation for establishing job security, which is then positively reflected in nurses’ job performance [33].

The findings of our study add to the growing body of literature evidence showing that various organizational issues, such as a rotation system, the availability of resources, the expectations of care staff, the provision of opportunities for continued professional development, and nursing autonomy, affect nurses’ achievement of their roles.

#### 4.2.1. Rotation System

Most of the participants mentioned the rotation system that Cyprus hospitals employ, and they described their personal journey in various, unconnected care settings. Job rotation entails strategically moving nurses across two or more hospital departments to improve their overall capabilities and benefit both nurses and hospitals [34,35]. The idea has historically been applied at the organizational level, and it was created to increase an organization’s flexibility and adaptability while giving workers credentials from different departments [36,37].

However, the findings of this study supported the notion that the rotation system caused feelings of annoyance, discomfort, and insecurity to nurses, strengthening previous studies. When implementing rotations, the frequency should be taken into consideration. This is because frequently having to work in different departments may result in nurses having reduced job satisfaction, caused by feelings such as unhappiness, insecurity, stress, frustration, fear, and anxiety, as well as feelings of exclusion, incompetence, and unwillingness [38,39,40].

Beyond the negative feelings expressed by respondents, one participant raised the issue of being bullied when their opinions did not coincide with those of the administration. Shorey and Wong [41] revealed that nurses were indoctrinated to keep their ideas to themselves by colleagues who held more powerful positions. Those who voiced their opinions were either subjected to disciplinary action or received unsatisfactory performance evaluations or unfair allocations. Given that professional happiness is linked to better performance, better organizational outcomes, and better quality of care, it is crucial to comprehend the relationship between nurses’ job rotation and their feelings of satisfaction [42].

The fact that participants voiced their frustration and insecure feelings about job rotations, and linked them with ineffective interaction with nursing administration, highlights the necessity for both the administration and the nurses to accept the changes. This outcome reinforces the study by Pinhatti et al. [40], which explored nurses’ feelings about a job rotation scheme among hospital units that was implemented to diminish conflict. They reported that nursing staff members had both positive and unfavorable feelings and perceptions. They concluded that job rotation is a proper management technique to minimize conflict, but they emphasized the necessity of including the staff before implementation.

On the other hand, very experienced nurses verbalized the positive outcomes of the rotation system, such as the expansion of knowledge, the acquisition of various nursing skills, and the prevention of professional burnout. Halberg et al. [43] agreed with these findings in a study that demonstrated personal advantages for job-rotating nurses of improved knowledge, abilities, and influence. Additionally, nurses experienced educational gains through exchanging knowledge. When this exchange occurred, they viewed job rotation favorably. Moreover, other studies [10,34,37,38,40] found that job rotation has positive effects on the following aspects: personal experience, development, and growth; improved motivation, knowledge, and skills; broader insight into organization and recognition of peers; a positive impact on job satisfaction; cultivation of collegial relations; and enhanced career opportunities.

Three very experienced participants who had been rotated only in CKD care settings claimed that rotation in related nursing settings enhances the knowledge base of the specific field and the development of particular nursing skills. They were employed in hemodialysis and peritoneal dialysis units, the nephrology ward, and the kidney transplantation unit. Two of them had twelve years of experience, whereas the third one spent twenty-five years in the nephrology ward and the hemodialysis unit. The literature supported the notion that job rotation that is based on the same category of patients can be a means of optimizing patient safety and quality of care by setting up a link between wards and unifying nursing care [43].

A rotation system would undoubtedly be advantageous to both nurses and organizations, but it should primarily be available to freshly registered nurses and nurses with some experience who may choose to advance their education in different fields. Furthermore, people who do not want to participate in rotations should always have access to permanent positions.

#### 4.2.2. Lack of Administrators’ Response to Deficiencies in Staff and Equipment

Nurses participating in the current study conveyed that hospital organizations and nursing administrations failed to address staffing and equipment deficiencies, and there was a lack of written guidelines for various nursing interventions. Nursing shortages are well documented in the literature [44,45]. Shortages have been linked to greater mortality rates of patients and more unfavorable patient outcomes according to numerous research conducted in various nations [46,47]. The issue is that, while many nations are challenged by inadequate staffing of nurses, multiple reports have focused on the standard of nursing care and the possibility that substandard nursing care could seriously harm patients [48,49]. When nurses have too many patients to care for, they are more likely to quit their jobs because they are fatigued and they do not have time to reflect or communicate with other nurses about various challenges [50].

The CKD care settings are considered technologically complex work environments where nurses deal with stressors associated with the nature of the work and their workplace surroundings, which can cause substantial rates of burnout. Due to the variety and unpredictability of CKD care work and the fact that CKD patients are considered difficult patients [51], nurses frequently report significant levels of workplace stress [52]. It is argued that nurses working in high-effort/low-reward conditions experience a hostile work environment that has a number of negative consequences on their attitude toward their work [53]. The effort–reward imbalance (ERI) model is widely used to explain job stress, and it has been applied to nurses in numerous nations [54,55]. ERI is linked to depression, poor health, and cardiovascular illnesses [56,57]. It might also result in a decline in workers’ job satisfaction and a rise in their intention to leave their current positions [58,59]. Additionally, this study found that there are not enough very experienced or expert nurses in CKD care settings in Cyprus hospitals due to the established rotation system, and that causes feelings of fear and insecurity.

The findings of the study also exposed the limited availability of equipment, especially hemodialysis machines. According to the respondents, the number of CKD patients has increased remarkably. Indeed, according to the Global Burden of Disease (GBD) [60], the global all-age prevalence of CKD has increased by 29.3% since 1990. The number of patients having renal replacement therapy goes beyond 2.5 million, and it is predicted to double to 5.4 million by 2030 [61]. Certainly, the lack of resources could lead to low standards of patient care. Inadequate physical resources and equipment, according to Blackman et al. [62] are predictors of missed care, whereas the availability of suitable modern equipment has a substantial impact on facilitating care delivery, lowering stress levels of nurses, and enhancing patient satisfaction. Furthermore, in a study by Rivaz et al. [63], participants recognized physical resources in the workplace, such as adequate and contemporary equipment, facilitate care and medical processes. Participants identified inadequate equipment as a significant factor that adversely impacted their work because they occasionally had to skip crucial care activities or they were delayed in providing care, both of which led to mental stress.

Based on the above, health organizations must reevaluate their priorities and find solutions to these issues, taking into account the fact that a shortage of nurses and lack of equipment are closely related to the reduced quality of care provided to patients, leading to serious implications for patients and nurses. Perkel [64] argued that good healthcare organization responds to the needs of patients and employees. Sevy Majers and Warshawsky [65] claimed that the foundation for using guided management decisions is laid out by nurse leaders who promote evidence-based nursing practice. However, the nurse leaders’ limited opportunities to be involved in the organization of healthcare results in a lack of confidence in their ability to make decisions under a scrutinizing and bureaucratic administration [66]. According to Honkavuo et al. [67], strong communication and long-term interaction with decision-makers enable caring policies that benefit patients to be applied in healthcare institutions. The driving force for such organizations to grow and change is found in nurse leaders’ ethical and real evolution, as well as their desire and willingness to progress and look to the future.

#### 4.2.3. Inconsistent Expectations of CKD Care Nurses

A very interesting finding is that nurse administrators have different expectations for the competencies that CKD nurses should have. The American Nurses Association and National Nursing Staff Development Organization [68] defined competency as “an expected and measurable level of nursing performance that integrates knowledge, skills, abilities, and judgment, based on established scientific knowledge and expectations for nursing practice” (p. 86). According to them, competence includes the qualities needed to function effectively in the nursing environment and facilitate high-quality, safe nursing care [69]. In this study, the nurse administrators’ expectations of CKD nurses are evidently incompatible. For instance, a nurse supervisor with long experience in the operating theater and only two years in the hemodialysis unit claimed that the ideal nurse is easy-going and disciplined and does not react negatively to the unusual time schedule of the hemodialysis unit. Additionally, the ideal nurse is quiet and, at the same time, very active. On the other hand, a different nurse administrator would expect nurses to always be patient, respectful, and compassionate with their patients. These inconsistent expectations are likely to lead to different goals in practice and, consequently, to significant shortfalls that prevent healthcare organizations from achieving their primary objectives and endanger healthcare quality, patient safety, and health outcomes [70]. Certainly, the quality of patient care is ultimately influenced by nurse administrators who have a major impact on workplace culture. They should clearly define the expectations for the employees and distinguish between safe and unsafe behavior.

#### 4.2.4. Opportunities for Continued Professional Development

It is generally recognized that nurses, including nephrology nurses, as healthcare professionals, must systematically update their knowledge and skills to meet the goals of their own practice. In a context of constant change marked by advancements in science and technology, as well as rising social and systemic expectations and needs, the necessity for nurses’ continuous professional development (CPD) increases. Nurses have not only the right but also a professional duty to engage in CPD [71], which is essential for keeping their knowledge current, their willingness to work, and their ability to provide patients with safe care [72]. CPD is defined as “a lifelong process of active participation by nurses in learning activities that assist in developing and maintaining their continuing competence, enhancing professional practice and supporting the achievement of their career goals” p. 1 [68]. It is evident from the literature that CPD encourages professionals to be enthusiastic, dedicated, and satisfied, which improves their retention and performance [73,74]. Because of the efforts to increase patient safety and decrease healthcare costs, all of these factors have an impact not only on nurses but also on organizations [75]. Self-motivation, relevance to practice, desire for workplace learning, strong enabling leadership, and a healthy workplace culture have all been cited as critical elements to enabling or optimizing the impact of nursing CPD in recent literature reviews [76].

However, the findings from this study suggested that respondents experience barriers to being present at such learning opportunities due to organizational factors or because they lack motivation. The participants emphasized how challenging it was to attend a nephrology nursing conference or seminar, that scientific journals were not available at work, and that there was a need for a nephrology nursing course.

The limited availability of time and a shortage of personnel were considered impediments to CPD, as these factors lead to scheduling problems when educational seminars or professional conferences are held [77]. Nurses’ lack of motivation to participate in continuous education programs has also been supported in the literature [78]. Additionally, other studies indicate that limited financial resources or the lack of an educational budget impact negatively on nurses’ willingness to participate in CPD programs [79]. Interestingly, one respondent in this study implied that conferences may not always result in desirable learning outcomes, which concurs with the work of Elsamian et al. [80], who found that conference agendas did not always align with educational demands.

According to our findings, although a specialist training course for CKD nurses would be important, it seems that the nursing administration has a different opinion because any professional development or acquisition of further academic qualifications is not considered for nurses’ promotion. Research indicates that professional development is not always acknowledged in the present, fast-evolving healthcare environment [81], and nurses engage in CPD when there are strong reasons for them to do so [82]. Therefore, it is crucial that strategies used to encourage nurses to participate in CPD address their actual needs [78]. However, as noted by Jho and Kang [83], the strategies created to encourage nurses’ CPD sometimes fail to take these needs into account.

#### 4.2.5. Nursing Autonomy

An issue raised by the data is that of nursing autonomy. Respondents expressed their experiences where senior colleagues and medical staff did not allow them to assess and initiate activities, such as patient education, independently. This finding is in accordance with the literature, which suggests that the level of autonomy that nurses have depends on the context and other variables or limits, such as organizational regulations or personal and individual considerations [84]. The definition of nursing autonomy encompasses a variety of characteristics, and it has arguably been confused with other concepts that are related to it, such as independence, self-governance, and accountability [85]. Clinical autonomy and professional autonomy are two well-known categories of nursing autonomy. The clinical autonomy of nursing professionals who offer direct patient care, according to Oshodi et al. [86], refers to their capacity to go beyond standard practice and make decisions on individual patients’ care. Professional autonomy can be applied to both the nursing profession as a whole and to individual nurses. It has been viewed as taking part in decision-making for the care of particular patients, as well as, more generally, developing nursing care practices to increase the standard of nursing and patient safety [87].

The findings of our research add evidence to the literature supporting that nurses do not feel professionally autonomous. Baykara and Şahinoğlu [88] argued that only 6.7% of the nurses who responded to their study claimed to have professional autonomy; they also noted that this autonomy was mostly constrained due to the necessity of relying on doctors for nursing interventions and a high nurse-to-patient ratio. Varjus et al. [87] implied that one of the key components of professional status is autonomy, and they emphasized the crucial role of nurse managers in determining how to empower nurses and establish environments that support independent practice. Furthermore, they provided evidence of nurses’ job satisfaction, which is a crucial component of the work environment that enables nurses to perform better on the job. In addition, they showed that nurses who work in environments that value their autonomy exhibit greater job satisfaction, have lower rates of burnout, and are less likely to leave the field.

However, the literature identified barriers to nurses’ autonomy in the hospital setting, some of which are consistent with this study’s findings. These include a doctor’s influence over a nurse’s work, the lack of technical–scientific knowledge, physical and emotional exhaustion from work overload, an insufficient physical structure, a lack of supplies, adherence to prescriptions, and the nurse’s reliance on the doctor for some care and/or action [89]. It is noteworthy that a lack of professional autonomy could have a negative effect on both the quality of care provided to patients and the satisfaction of the nurses themselves.

### 4.3. Barriers to Patient Education

What was obvious from the findings of the current study is that there is no systematic patient education provided to CKD patients, although nurses perceive it to be a significant nursing role and also a fundamental aspect of patient care [90]. Patient education is a concept that encompasses informing patients about their condition, providing them advice and information, and showing them how to intentionally modify their behavior [91]. The concept of patient education also entails two-way interaction between the patient and the nurse and tries to develop and improve health or teach people how to adapt to conditions [92]. Patient education has been defined as a planned interactive learning process designed to support and enable people to manage their life with a disease and optimise their health and wellbeing [93]. It can result in a variety of beneficial health outcomes, such as enhancing patients’ understanding of their condition and adherence to treatments, as well as their quality of life and ability to manage it. According to Aghakhani et al. [25], patient complaints are most frequently linked to a lack of education about their condition, whereas Bennett [94] identified patient education as one of the key determinants and characteristics of high-quality nursing care.

Our findings strengthened the body of data in the literature by outlining a number of factors affecting the implementation of nursing activities by nursing staff.

#### 4.3.1. Inadequate Administration Guidance

The majority of participants reported that they do not educate their patients because nursing management and specifically the head nurses or nurses in charge of their units do not provide them with any direction or support. They clearly related the presence of a policy for patient education in daily nursing practice with nurses regularly implementing patient educational activities. Nurse managers are responsible for setting standards, approving content, and assigning time for nurses to educate patients [95]. The aforementioned findings of our study are consistent with the work of Fereidouni et al. [96], who demonstrated that administrators did not sufficiently supervise the patient education process and did not prioritize it. Additionally, they observed that the supervisors’ practical engagement was insufficient, and they did not prioritize patient education during their rounds or pay attention to it. Likewise, Daly et al. [97] concluded that nurse managers must become more involved in clinical leadership and supervise clinical care if nursing quality and safety are to be improved.

In a study by Armstrong et al. [98], which aimed to explore whether nursing unit managers’ actions promoted the delivery of high-quality patient care, it was found that direct patient care accounted for 25.8% of unit administrators’ time, which was more than other tasks, such as administration, communication, and patient support. Providing direct care to patients includes positioning patients, helping new mothers breastfeed, handing out analgesics, or helping patients eat. The nurses in charge remarked that, rather than giving these fundamental tasks of nursing care to a more junior nurse, they felt obligated to perform them since there was no other option, either because the task was difficult or because of a lack of staff. Prezerakos et al. [99] indicated that the head nurse’s insufficient authority and the director of nursing’s ignorance were barriers to hemodialysis patient care and claimed that one of the most crucial elements in the workplace is the capacity for the director of nursing to create a framework for the provision of high-quality nursing care.

Effective nurse leaders are crucial for clinical practice because they make sure that the necessary personnel and resources are in place to deliver safe care and the best possible patient outcomes [100]. According to a study by Bennett [94], nephrologists, hospital or health center managers, and head nurses all impact the quality of nursing care. The head nurse and the hospital management have the biggest influence. According to patients, the ward manager, nurses, and outside influences could affect how well hemodialysis patients are cared for.

#### 4.3.2. Shortage of Staff and Limited Time

Even though nurses consider patient education a practice that is inextricably linked with quality patient care, the condition of work overload due to the shortage of staff and limited time is a challenge to patient education. The findings add evidence to the literature that consider these issues to be the main barriers to various nursing interventions [44,45,101], including patient education [25,102]. A large study carried out by Bennett et al. [103] in 20 European and three Asian countries aimed to identify potential barriers and enablers of rheumatology professionals to apply the European Alliance of Associations for Rheumatology (EULAR) recommendations for patient education. The lack of time was the most frequently mentioned barrier to including patient education in routine patient care. Although the participants in this study believed that it was beneficial to meet the needs of the patients, they perceived it as additional work. Furthermore, it was concluded that several tasks, such as patient education evaluation, were not always given priority due to time constraints. Additionally, Nikitara et al. [101] highlighted the lack of time as one of the most commonly mentioned challenges nurses have when conducting their tasks, specifically for educating patients.

A lack of staff was also included as a barrier. It was found that patient education was frequently not available because no one on staff, such as skilled nurses, had specialized knowledge. According to Ball et al. [104], due to inadequate staffing levels, the most frequently neglected tasks nurses reported were communicating with patients and educating them. Further, patients also consider that time restrictions and staff shortages were barriers to their adherence to treatment because they felt discouraged from asking questions, trying to obtain more knowledge, and developing necessary skills [105]. In addition, patients perceive nurses working in a hemodialysis unit to become exhausted, as there are too few to handle the tremendous workload, and there is a lack of resources [106].

Numerous research projects have demonstrated that a lack of nurses is linked to increased mortality rates among patients and an increase in adverse patient outcomes [46,47]. In parallel, governments around the world are attempting to address the challenge of lowering healthcare costs while maintaining the quality and safety of healthcare systems [104]. In a systematic literature review studying nursing shortages and identifying the reasons why nurses decide to leave the profession, it was concluded that nurses are more likely to quit their jobs as a result of feeling overwhelmed from caring for more patients. As well, nurses were too busy to reflect on their actions or talk to their teammates about them [50]. Interestingly, a participant in our study confirmed the interrelation between patient education and the availability of time. He stated that he had time for patient education because he was working in a small hemodialysis unit at a provincial hospital that had only six dialysis machines.

#### 4.3.3. Limited Knowledge

The findings of our research add to the body of evidence that many nurses working in CKD care settings lack basic knowledge about caring for and educating nephrology patients. Participants clearly verbalized their restricted and superficial knowledge to meet the learning needs of CKD patients. Even very experienced nurses characterized their knowledge as inadequate to teach patients about the disease, their diet, hemodialysis machines, etc. Nurses’ lack of knowledge regarding CKD care, including patient education, has been well documented in the literature [107,108,109,110,111,112]. Nobahar and Tamadon [27] found that the focus should be on nurses’ basic knowledge as part of the qualities and skills they must possess in order to deliver quality patient care.

The results of our study are in line with the findings of Greer et al. [110], who implied that a lack of adequate knowledge or skills is one of the main obstacles to educating patients about CKD and related therapies. In addition, concerns about emotionally exhausting patients, time restrictions for treatment sessions, a lack of reimbursement for CKD education, and a lack of educational tools were also highlighted as obstacles.

In the study by Matthews and Trenoweth [113], nurses highlighted the importance of self-management, but some of them seemed not to understand this term fully. Additionally, they did not provide details concerning how they support patients in self-managing their condition. Likewise, the findings of another study imply that, while many nurses may not be familiar with the idea of health literacy, they employed a variety of tactics to make sure their patients understood the information being provided. There were no apparent strategies at the organizational level to educate front-line employees on the concept of health literacy, measure health literacy, or engage groups or the community to improve health literacy [114].

Patient education evidently results in many benefits for patients, such as better knowledge, quality of life, and self-care, as well as fewer readmissions to the hospital and better medication compliance [115,116]. Once individuals are made aware of CKD, they may adopt a healthy lifestyle that reduces the risk of morbidity and mortality [117]. Although education benefits patients, nursing personnel, and health organizations [118], nurses do not feel competent enough to be successful educators and need support to do so [119]. Educating patients efficiently entails knowledge and skills. Our respondents attributed their limited knowledge of patient education not only to their lack of preparation during their studies but also to the absence of a specialty nephrology program. These findings concur with the literature, supporting the notion that nurses can improve their educational capabilities with proper education and training [119,120]. Nurses’ ability to educate efficiently can optimize patients’ knowledge, skills, self-care management competencies, and power to make informed choices [120,121]. Patient education is perceived as a practice that requires nurses to actively involve patients in order to meet their specific learning needs. Even though implementing effective and efficient patient education activities is difficult in busy healthcare settings, it is possible [122].

#### 4.3.4. Nurse–Doctor Boundaries

One of the primary challenges confronting healthcare organizations is how to ensure the continuous provision of high standards and safe care of patients in a dynamic multiprofessional environment. Care is provided by a variety of people and groups from various professions, all of whom share a strong awareness of their respective identities, relative status differences, and boundaries within and between the various professions. These people and groups all have unique cultures, identities, educational backgrounds, objectives, bonuses, and incentives [123,124]. In addition to being aware of these disparities and boundaries, health professionals also view developing, upholding, and defending them as an integral part of their daily work lives [125]. Hence, professional identities and boundaries have a big impact on how people interact with counterparts in their discipline and in other disciplines, which has an impact on the treatment that patients experience.

Our findings revealed that doctors coerce nurses into abstaining from patient education and remaining at the perimeter of physical treatment. Our respondents emphasized that, although nurses may be knowledgeable about patient education, they do not have the authority to do so because, where their professional jurisdiction stops, the doctors’ jurisdiction begins. This is corroborated in several studies highlighting the constraints of nurses’ autonomy due to their traditionally subordinate role to physicians, which is connected to poor cooperation and nurses’ uneven positions in the working community [126,127]. The lack of respect for or disregard for nurses’ knowledge and competence [128,129,130], and related inequalities in their positions within the workplace, including nurses’ subordination to doctors [131], have long been evident.

It was obvious from the findings of our research that nurses hesitated to step out of the doctor–nurse relationship, indicating no clarity between the nurses’ and doctors’ responsibilities. In addition, they seemed to be reluctant or even terrified to cross any boundaries even though patient education has long been considered a mandatory and independent nursing role that meaningfully impacts patients’ health and quality of life. In order to counter this, senior leadership of healthcare organizations should encourage nurses to be equal members of care teams. The clinical autonomy of staff nurses who offer direct patient care, according to Oshodi et al. [86], refers to their capacity to surpass accepted practice and make decisions about individual patients’ treatment. Nurses’ independence in the era of decision-making could result in remarkable nursing outcomes [126,132] and higher work quality [133].

Based on the aforementioned literature outcomes, we could draw the conclusion that nurse leaders should establish and uphold workplaces where nurses are aware of their expectations and responsibilities, which do not alter despite the circumstances. Additionally useful are permanent plans for nurses’ professional development and clear job descriptions for nurses [134]. A safe work environment, a welcoming and tranquil culture, good team spirit without disagreements or taunting, and well-established unit protocols to follow are reported to improve nurses’ autonomy [129,135].

### 4.4. Difficult Patients

Patients diagnosed as non-compliant are always stigmatized when they are described as being recalcitrant, deviant, manipulative, failures, cheats, and rule-breakers, among other epithets [136]. The term “difficult patient” encompasses a negative attitude solely toward the patient. According to Duxbury [137], difficult patients are those who make nurses feel frustrated, uncomfortable, or ineffective. Nurses often use the term “difficult” to describe a range of non-compliant behaviors that patients demonstrate, such as self-harm and aggressive, demanding, attention-seeking, dependent, splitting, deceptive, manipulative, and disinhibited behaviors [138]. Macdonald [139] reported that nurses inappropriately label patients, which can lead to a global view of a patient that compromises care.

#### 4.4.1. Self-Neglect

It was reported in our interviews that some patients give up on themselves and made it apparent that it was their own responsibility that they had progressed to the point of end-stage renal disease and required renal replacement treatment. It was evident that nurses felt that patients who have a chronic ailment, such as diabetes mellitus, high blood pressure, or any other condition that caused chronic kidney failure, should be directly responsible. According to the evidence, the participants found that these patients’ current health difficulties were attributable to their own negligence, which resulted in inadequate management of their diabetes mellitus, failure to control their blood pressure, or any other health issue.

As defined by societal norms, self-neglect is the incompetence or unwillingness to meet one’s own essential needs [140]. According to a widespread definition in the literature, self-neglect is the inability to meet one’s own basic needs or behave in a way that threatens that person’s self-care [141]. Poor hygiene, home degradation, hoarding, poor nutrition, social withdrawal, service rejection, not taking medication, endangerment behaviors, a lack of shame, and other characteristics are frequently listed as signs of self-neglect [142].

Practitioners frequently observe it in relation to conditions of aging, such as frailty, which are marked by a person’s losses in the social, psychological, and physical realms. Adult Protective Services (APS), a national agency tasked with looking into the abuse, neglect, and exploitation of vulnerable persons, receive reports of self-neglect most frequently [143]. Corresponding to other research, older individuals who self-neglect have a death rate that is 1.5 times higher than adults who do not self-neglect [141]. Furthermore, those who self-neglect have a death rate that is up to six times higher after one year, and they are fifteen times more likely to die [144].

For a number of reasons, depression is a particularly salient risk factor for self-neglect. First, research has demonstrated that depression is an independent risk factor for a range of actions that could indicate self-neglect, such as dietary indiscretion and non-adherence to medication [145]. A type of depression that includes executive dysfunction, the capacity to organize and sequence tasks, is linked to activities of daily living (ADL) impairment that is not proportional to the severity of mood disorder [146]. This is so remarkable, considering that about half of the populations of two countries, 133 million Americans and 16 million Canadians, live with at least one chronic condition, and one in four experience limitations on daily activities as a result [147]. When a chronic condition, such as chronic kidney failure, is diagnosed, people are faced with a number of responsibilities that are important for both physical and psychological adjustment. For the patient to manage the symptoms of the illness or cope with the demands of treatment, accepting the disease, learning new skills, and making modifications to daily routines are more than necessary.

#### 4.4.2. Negative to Instructions and Learning

Given that CKD is a chronic disease, patients are generally treated with long-term regimens including renal replacement therapies, medications, diet, fluid restriction, physical management, etc. To successfully manage their illness, avoid and control acute and chronic consequences, and improve their quality of life, CKD patients must continuously engage in self-care [148]. Self-care is frequently difficult because it calls for patients to maintain strict self-control for the rest of their lives. [149]. The term self-care refers to a broad concept that incorporates treatment adherence, as well as more proactive self-care actions; poor treatment adherence is viewed as a limited form of passive self-care [150]. Treatment adherence refers to the treatment recommendations made by a health professional, and it is critical to all CKD patients’ health management. It includes the replacement kidney treatment, including fluid and dietary restrictions, and taking medicines [151].

However, the findings of my research highlighted that some CKD patients do not adhere to certain advice and instructions from nurses. It was found that patients do not want any instructions or further information because they claim to already know everything, and in certain cases, they react by using strong language. One participant referred to her experience when she was trying to inform one of her patients about fluid and food restriction. The patient actually challenged the nurse by saying, “I don’t drink water”. That caused the nurse to characterize her as a “stubborn woman”. Certainly, these behaviors point out patients’ strongly negative attitudes about listening and following detailed information or orders, and they generally show an unwillingness to accept and adhere to treatment.

Adherence discrepancy is brought on through a number of intricate behavioral traits among patients, such as their health beliefs, self-efficacy, unfavorable nurse–patient relationship, and therapy acceptance [152,153,154]. The important correlation between CKD patients’ acceptance and treatment adherence is clear in the literature that supports the idea that patients who accept the diagnosis and treatment suggestions tend to adhere to them more closely [155]. When patients are unable to alter the situation and lessen their dependence on the treatment, they accept the treatment and their condition. The patients typically move on and embrace their new situation by obeying the advice of the healthcare professionals [156].

A factor that contributes to patients’ unwillingness to follow instructions and adhere to treatment is the inability of CKD patients to acquire and comprehend fundamental information related to their health condition. A literature review implied that 9% to 32% of CKD patients may have limited health literacy [157], which contributes to poor patient self-management [158]. Health literacy is defined as “the degree to which individuals have the capacity to obtain, process, and understand basic health information needed to make appropriate health decisions” [159]. Moreover, the burden of their disease, in addition to severe symptoms or functional impairment caused by other health conditions that lead to frequent fatigue and chronic pain, has a negative impact on patients’ willingness to learn and follow instructions [160].

#### 4.4.3. Denying Reality

Our findings revealed that nurses report that a number of patients do not accept their health status and consequent treatment, and as a result, they withdraw into themselves. Acceptance can help a person cope with unavoidable negative events that occur by maintaining their psychological wellbeing and ability to act. It is the ability to face reality even when it does not align with one’s expectations or desires and the readiness to deal with it in any case [161]. Carver et al. [162] asserted that such people “make every effort to stay engaged with the important goals that give structure to their lives” (p. 387). It is expected that, when patients accept their illness, they will modify their life goals to more realistic ones by incorporating this challenging life event [161]. Chronic illnesses are viewed as life events that bring a person’s psychological balance down, and acceptance is essential to reestablishing that balance. Patients with chronic pain and fatigue report higher levels of depressive moods and decreased mental health-related quality of life (MHQL) when they refuse to accept their medical condition and employ an assimilative coping technique. In the CKD population, adaptation strategy is linked to compliance [163], and avoidant coping has been linked to mortality in individuals with end-stage renal disease [164]. In a cross-sectional questionnaire study by Poppe et al. [165], it was found that acceptance was correlated with improved physical and mental health quality of life in a group of CKD patients. A crucial goal in the treatment of CKD patients is the improvement of health quality of life (HQL), and it is considered to be dependent on adaptive coping, which includes cognitive and behavioral efforts to manage stressful conditions or associated emotional distress [166]. Undoubtedly, CKD is a complicated, progressive condition that forces patients to constantly adapt to changes in medications, treatment plans, and behavioral patterns while becoming more and more dependent on medical technology and their surroundings. Acceptance of the disease and flexibility in rearranging their lives are prerequisites for adjusting well to these evolving treatment modalities.

#### 4.4.4. Being Angry

These findings add evidence to the literature highlighting that nurses are one category of employees who face the risk of confronting verbal or physical aggression in their workplace [167,168]. Aggression, an intrinsic, deeply emotional force, is difficult to define, as it is inherently context dependent [169]. Based on certain disciplines, aggression can be categorized as an offensive or defensive force in biology science, impulsive or premeditated in the study of crime and criminal behavior, and reactive or spontaneous, emotional, or instrumental in behavioral psychology [169]. Rippon [170] suggested a definition that includes the following elements: (a) intent (behavior intended to harm another living being); (b) expression (physical or verbal, emotional or psychological, active, or passive); and (c) emotional state (it can happen along with emotions such as anger, or with no emotion). Aggression could be socially acceptable, adaptive behavior. For instance, patients might demonstrate aggressiveness by defending their rights. The aggressive behavior in this instance might not violate another person’s boundaries, rules, or standards [171]. Another type of aggression takes place when patients physically attack, verbally insult, and threaten healthcare professionals. Worldwide, 60% of nurses report having encountered verbal or non-physical aggression, and 30% have experienced physical violence [172]. All healthcare settings are impacted, despite the fact that the majority of aggressive incidents occur in mental health departments, as well as in accident and emergency departments [173]. 

Patient aggression has a detrimental effect on staff morale, the effectiveness of healthcare organizations as a whole, and the quality of patient care. It is evident that it lowers the standard of patient care because it disrupts unit operations, results in treatment errors, creates delays in task completion, and lengthens patient wait periods [174,175]. Furthermore, aggression decreases job satisfaction and motivation among nurses and may cause stress in addition to a rise in staff turnover and early retirement from the nursing field [176].

The respondents expressed that anger, irritation, and aggressiveness of their CKD patients are common behaviors, which is confirmed by the relevant literature. According to Jones [177], disruptive, violent, and aggressive behavior by patients and occasionally by their family members is becoming a serious issue in some hemodialysis units. Sedgewick [178] surveyed nephrology nurses in the United Kingdom (UK) and reported that 80% of participants experienced some sort of violence or aggressiveness at work over twelve months. Waiting for treatment, traveling times and costs, poor communication with nursing staff, feeling no active participation in or control over their treatment, and mental health issues have been identified as the main causes of CKD patients’ aggression [179]. According to Kurella et al. [180], the prevalence of cognitive impairment and dementia is greater in individuals with end-stage renal disease than it is in the general population.

Interestingly, one of our study participants blamed such patients’ behavior on nurse management since they actually allow patients to behave in an indiscipline and aggressive manner. Nurse managers are essential for creating supportive, safe, and low-aggression workplaces [181,182]. However, research findings indicate that nurse managers may underreport or overlook patient aggression and staff protection in order to prioritize customer courtesy or to maintain a positive public image [183,184].

### 4.5. Nurses’ Defensive Behavior

The findings of this study clearly showed that nurses who have been confronted with difficult patients and stressful situations frequently exhibit defensive behaviors in an attempt to prevent emotional overload and exhaustion. Nurses’ protective behaviors involved maintaining a distance and displaying disinterest. Smith and Hart [185] explained how nurses refer to patients as “difficult” when their behavior makes it difficult for them to maintain emotional control, whereas Sheldon et al. [186] clarified that negative feelings might make communication difficult between a nurse and a patient.

Menzies [187] first studied the high level of stress in hospitals and supported that anxiety is connected to primitive concerns aroused in the nurse by contact with seriously ill patients. That study elaborated on defensive techniques that nurses could employ to cope with anxiety while providing care. The first recommended technique is to “split up the nurse–patient relationship”. It supports the notion that the main cause of anxiety for nurses is the relation with the patient. The closer nurses get to their patients emotionally, the more likely they are to experience anxiety. In order to afford themselves some protection from anxiety, they perform a limited number of tasks, repeatedly, and have limited contact with the patient. Menzies implied that feelings of anxiety are the fundamental roots of distorted or alienated relationships at work.

Khalil [188] examined violence in nursing using a qualitative approach. The employed questionnaire asked respondents to share experiences of specific incidents of violence within the nursing profession, especially in the context of “good” and “difficult” patients. It was found that “good” patients were rewarded with tender, loving care, although difficult patients were ignored, or the interventions they needed were deliberately delayed. Although most nurses provided the most appropriate nursing interventions for the patients irrespective of their behavior, time pressure and nursing staff shortage made them more likely to categorize the patients into “good” and “difficult”, in order to protect themselves from traumatic encounters with “difficult patients”.

In addition, Michaelsen [189] studied nurses’ relationships with difficult patients and identified three different strategies for nurses’ behavioral and emotional reactions: “persuasion”, “compromise”, and “avoidance”. The persuasion strategy was defined by nurses’ beliefs that patients would accept advice and directions and their efforts to motivate the patients to comply. This could be accomplished in a variety of ways, from offering advice to making threats. The compromise strategy was characterized by the nurse finding a compromise between using persuasion and avoidance. The avoidance strategy was defined as the nurse keeping the patient at a greater emotional distance by withdrawing either physically or psychologically. The avoidance strategy resulted in nurses not recognizing important social and health problems of some patients and some nurses who used it expressing a fear of losing contact with their emotional lives.

The findings of Ross et al. [190] highlighted that nurses working in hemodialysis units are at risk of experiencing a sense of failure and powerlessness as patients’ mental or physical health deteriorates, or they may become frustrated with non-compliant or aggressive patients. The researchers argued that it is difficult to manage a patient who may be aggressive. The inability to cope with this stressor can cause nurses to become critical of themselves. In the long term, having to cope with stressors impacts nurses’ psychological wellbeing, social functioning, and somatic health, and it causes illness. They supported the notion that the coping technique of distancing is not usually chosen by nurses, and an assumption can be made that the majority of nurses do not minimize an event or detach themselves from work-related stressors.

## 5. Study’s Strengths and Limitations

The study has offered a clear and thorough explanation of its rationale and content. Participants’ perspectives and experiences were explored in depth and contextualized with the relevant literature. The sample size of 16 is appropriate for qualitative research, particularly within the interpretative phenomenological analysis (IPA) framework. To support data saturation and enhance the richness and transferability of the findings, rigorous methods were employed. Limitations include the single-country context (Cyprus), possible response bias due to one of the researchers being a well-known figure within the local nephrology nursing community, and the exclusion of other stakeholder perspectives. Still, nurses’ insights meaningfully enhanced the understanding of their experiences in CKD care.

## 6. Conclusions

This study contributes significantly to the limited literature on the roles of nurses in chronic kidney disease (CKD) care, with a focus on the Cypriot healthcare context. Despite the essential contribution nurses make to the management of CKD, their roles remain poorly defined, inconsistently supported, and underdeveloped. The findings demonstrate that nurses deliver a broad spectrum of care—ranging from technical interventions to psychosocial support—yet systemic barriers prevent them from reaching their full potential.

A major theme emerging from the research is the insufficient preparation nurses receive in renal care. Most participants reported feeling unprepared, citing a lack of targeted training during undergraduate education and minimal opportunities for specialized professional development. Organizational factors further compound these challenges. The job rotation system, lack of administrative responsiveness, unclear role expectations, and limited clinical autonomy were identified as key constraints. These issues have been shown to negatively impact nurses’ confidence, motivation, and capacity to deliver high-quality, consistent care.

Another critical barrier is the absence of structured patient education, with participants pointing to time limitations, inadequate support, and professional boundaries that restrict their educator role. The emotional toll of working with CKD patients—many of whom struggle with adherence and present with challenging behaviors—also contributes to nurses adopting defensive strategies, such as emotional withdrawal, as a form of self-preservation.

The study’s outcomes underscore the urgent need for a strategic and systemic response to enhance the nursing role in CKD care. To address this gap, a framework has been developed to support role clarity, educational advancement, and structural alignment within healthcare institutions. Implementing this framework could lead to improved patient outcomes, including reduced complications and hospital admissions, better quality of life, and more cost-effective care delivery.

Ultimately, clarifying and expanding the role of nurses in CKD care is not only a professional imperative but also a public health priority. With appropriate preparation, institutional support, and recognition, nurses can play a pivotal role in transforming the quality and sustainability of CKD care services both in Cyprus and internationally.

## Figures and Tables

**Table 1:** Nurse participants in interviews.

N	Sex	Age	Years of Experience	Hospital	Hospital CKD Services
1	Male	25	1	3	Hemodialysis
2	Male	31	6	5	Hemodialysis
3	Female	32	3	2	Hemodialysis/Peritoneal/Nephrology Ward
4	Female	27	5	4	Hemodialysis/Peritoneal Dialysis/Nephrology Ward
5	Female	45	14	4	Hemodialysis/Peritoneal Dialysis/Nephrology Ward
6	Male	41	1	2	Hemodialysis/Peritoneal/Nephrology Ward
7	Female	33	12	1	Hemodialysis/Peritoneal Dialysis/Nephrology Ward/Transplantation Unit/Autoimmune diseases
8	Female	33	12	1	Hemodialysis/Peritoneal Dialysis/Nephrology Ward/Transplantation Unit/Autoimmune Diseases
9	Female	34	9	5	Hemodialysis
10	Male	35	16	4	Hemodialysis/Peritoneal Dialysis/Nephrology Ward
11	Female	45	21	1	Hemodialysis/Peritoneal Dialysis/Nephrology Ward/Transplantation Unit/Autoimmune Diseases
12	Female	34	15	4	Hemodialysis/Peritoneal Dialysis/Nephrology Ward
13	Female	55	29	4	Hemodialysis/Peritoneal Dialysis/Nephrology Ward
14	Female	36	14	2	Hemodialysis/Peritoneal/Nephrology Ward
15	Female	47	25	2	Hemodialysis/Peritoneal/Nephrology Ward
16	Female	52	29	5	Hemodialysis

## Data Availability

The data sets used and/or analyzed during the current study are available by the corresponding author upon reasonable request.

## Abbreviations

The following abbreviations are used in this manuscript:

ACR	Albumin–creatinine ratio
CKD	Chronic kidney disease
CV	Cardiovascular
eGFR	Estimated glomerular filtration rate
ERA-EDTA	European Renal Association
ESKD	End-stage kidney disease
GBD	Global burden of disease
GESY or GHS	General health system
GP	General practitioner
HF	Heart failure
KDIGO	Kidney Disease: Improving Global Outcomes
LMICs	Low- and middle-income countries
PRISMA	Preferred Reporting Items for Systematic Reviews and Meta-Analyses
RN	Registered nurse
RRT	Renal replacement therapy
SHSO	State Health Services Organisation
